# Supplementary material for: Identification of conserved frontal neurophysiological markers of cognitive flexibility in humans and rats
Source: Commun Biol. 2025 Aug 23;8:1268. doi: 10.1038/s42003-025-08729-x (PMC12375130; doi:10.1038/s42003-025-08729-x)
Supplement: Supplementary file 2 — Supplementary Material [file 42003_2025_8729_MOESM2_ESM.pdf]

Supplemental Materials

Supplementary Methods

Q-learning modeling of PRL behavioral data and quantification of model performance

We fit a variety of Q-learning models to the behavioral data. On each trial (t), the value (Q) of the chosen action (c) is estimated according to the outcome delivered (r; reward = 1, no reward = 0). Q values were initialized to 0.5 (neither good nor bad since the target and non-target stimuli were randomly assigned at the beginning of the session). Prediction errors (PE) — the difference between the estimated value of the chosen action (Q_c) and the actual outcome — were computed according to:

$$PE = r_{(t)} - Q_{c(t)} \tag{1}$$

The PE was then used to update the value estimate for the chosen action (but not the non-chosen action) on a trial-by-trial basis according to equation 2.

$$Qc_{(t+1)} = Qc + \alpha \times PE \tag{2}$$

The α parameter is a learning rate, ranging from 0 to 1, that controls how quickly the PE updates value estimates. A low α results in Q-values that gradually changed over multiple trials, whereas a high α causes more rapid changes in Q-values.

The above equations are used to modify the value attributed to each action. The values are used to guide choices by converting them into action probabilities using the softmax equation.

$$p(A) = \frac{e^{QA \times \beta}}{e^{QA \times \beta} + e^{QB \times \beta}} \tag{3}$$

The degree to which choices are exploratory (i.e., selecting the lower-valued action) vs. exploitative (i.e., selecting the higher-valued action) is controlled by the inverse temperature (β) parameter. A higher β parameter indicates a greater tendency to engage in exploitative choices

whereas a lower β parameter is indicative of a greater tendency to explore actions associated with a lower value. The model above describes the simplest model that we evaluated and includes two free parameters (α, β) that are estimated.

We implemented several modifications to determine if any model variant provided a better explanation of behavior. As one example, PRL performance might involve simultaneously updating the value of both the chosen and the non-chosen actions after each PE. To evaluate this possibility, we implemented a model with a “double update” rule, whereby the value of both the chosen and unchosen actions are updated by the PE[1]. The value of the chosen action is updated according to equation 2 but the value of the unchosen action (Qnc) is updated according to:

$$Qnc_{(t+1)} = Qnc - \alpha \times PE \tag{4}$$

With this variant, a rewarded left response would increase the value attributed to the left action while simultaneously decreasing the value associated with the right action. Alternatively, another approach that modulates the value of both actions on the same trial incorporates a method to decay the value of the unchosen action. This approach is designed to capture “forgetting” the value of an action if it has not been selected for several trials[2,3]. Again, the value of the chosen action is updated according to equation 2, but the value of the unchosen action is updated according to:

$$Qnc_{(t+1)} = (1 - forget) \times Qnc \tag{5}$$

When the forget parameter equals zero, then the value of the unchosen action is unaffected, and the model is identical to that described by equation 2. By contrast, if the subject chooses the left action and the forget parameter equals 0.5, then the value of the right action is reduced by 50% for each trial where the left action was selected.

Each of the model variants above were used in conjunction with the softmax function (described by equation 3) to convert value estimates into choice probabilities. Although the

inverse temperature (β) parameter regulates the degree to which the subject engages in explorative vs. exploitative decisions, choice behavior could also be influenced by the presence of a bias for one action or another. Therefore, rather than using the standard softmax function (equation 3), each of the model variants described above can also be used in conjunction with a modified softmax function that includes a bias parameter:

$$p(A) = \frac{e^{(QA+bias) \times \beta}}{e^{(QA+bias)} + e^{QB \times \beta}} \tag{6}$$

A bias parameter of 1 would indicate an extreme bias for action A and a bias parameter of -1 would indicate an extreme bias for action B. When the bias parameter was 0 then the subject exhibited no bias for either action and equation 6 would function in a similar manner to the softmax function described in equation 3.

The models described above all used a single α parameter to control the rate at which PEs updated value estimates. To explore whether a difference in the sensitivity for rewards vs. non-rewards influenced PRL performance, we also implemented a series of models that used separate learning rates for each outcome, as follows:

$$Qc_{(t+1)} = Qc + \alpha - gain \times PE \; if \; reward = 1 \tag{7}$$

$$Qc_{(t+1)} = Qc + \alpha - loss \times PE \; if \; reward = 0 \tag{8}$$

If the response resulted in a reward, then the rate at which PEs updated value estimates was controlled by the positive learning rate (α-gain; equation 7). By contrast, for non-rewarded trials a negative learning rate was used (α-loss; equation 8).

Using the equations described above, we fit 10 separate models to the PRL data. The optimal parameter values for each of the models were identified by minimizing the negative log-likelihood of choice probabilities using the “minimize” optimization function with the L-BFGS-B algorithm in Python’s Scipy library (v.1.5)[4]. To sample broadly from the range of possible

parameter values and reduce the possibility of the model getting stuck in local minima, the optimization algorithm was initialized at five different starting points within the parameter space for each subject. The parameter values associated with the lowest negative log-likelihood were selected as the best-fitting set for each subject.

The best-fitting model was determined by comparing the Bayesian Information Criteria (BIC) value for each model. The ∆BIC value was calculated using the difference between the BIC of each model from the lowest BIC value and used to calculate the relative likelihood as follows:

$$relative\ likelihood = e^{(-0.5 \times \Delta BIC)} \tag{9}$$

To confirm the validity of the best-fitting model, we conducted posterior predictive checks in which we compared the simulated performance with actual PRL performance for human and rodent subjects. Parameter recovery exercises were also performed to confirm the accuracy of parameter estimation[5].

To determine the relationship between the ERPs and reward PEs, we used generalized linear models to predict the trial-by-trial neural activity at each timepoint within a trial. The first regression model presented in Figure 3 included the reward PE for each trial as the sole model predictor whereas the regression model presented in Supplemental Figure 5 included the outome of the trial (i.e., rewarded or not) and the value of the chosen action (i.e., the expected value). This approach is designed to delineate between error signaling and outcome valence[6,7]. In both models, neural activity for each timepoint within the trial epoch was used as the dependent variable. To predict the ERP waveform for a given reward PE value, we multiplied a hypothetical reward PE value (e.g., -0.6, -0.3, 0.3, or 0.6) with the regression coefficient and added the model intercept. This step was repeated for each timepoint.

Supplementary Tables

Supplementary Table 1. Quantification of Model Performance

Model performance was quantified and evaluated using BIC. The best-fitting model with the lowest BIC value for both species incorporated a single alpha learning rate and forget parameters (oaf). o=one; t=two; a=alpha; f=forget; du=double update; b=bias.

	Humans			Rats		
**Model**	**BIC**	**Δ BIC**	**Relative Likelihood**	**BIC**	**Δ BIC**	**Relative Likelihood**
oaf	239.963	0	1	341.830	0	1
tadu	240.117	0.154	0.92589	344.949	3.119	0.210241
oa	242.149	2.186	0.335209	345.204	3.374	0.185074
taf	243.634	3.671	0.159534	346.296	4.466	0.107206
oabf	244.164	4.201	0.122395	349.840	8.010	0.018224
oab	244.515	4.552	0.102694	351.546	9.716	0.007766
ta	244.852	4.889	0.08677	352.845	11.015	0.004056
oadu	246.246	6.283	0.043218	354.768	12.938	0.001551
tab	247.628	7.665	0.021655	355.848	14.018	0.000904
tabf	247.810	7.847	0.019772	357.235	15.405	0.000452

Supplementary Table 2. Effect of modafinil on PRL performance

In humans, modafinil treatment had no significant effect on the number of completed reversals, or win-stay or lose-shift responses for either response. By contrast, there was a significant dose on the number of completed reversals when rats were administered modafinil [$F(5,45)=3.17$, $p<0.05$], which was due to a reduction when administered 64 mg/kg ($p<0.05$). Similarly, a dose effect was evident when target win-stay responding was analyzed [$F(5,45)=3.05$, $p<0.05$] that was also driven by a reduction in the highest treatment group ($p<0.05$). Modafinil treatment had no significant effect on the remaining PRL measures.

	**Humans**				
**Dose**	**Completed Reversals**	**TWS**	**NTWS**	**TLS**	**NTLS**
**Placebo**	4.01 ± 0.30	0.88 ± 0.01	0.82 ± 0.04	0.58 ± 0.04	0.67 ± 0.03
**Low**	4.40 ± 0.39	0.87 ± 0.01	0.84 ± 0.04	0.55 ± 0.04	0.67 ± 0.03
**High**	4.39 ± 0.30	0.88 ± 0.01	0.85 ± 0.03	0.52 ± 0.04	0.66 ± 0.03
	**Rats**				
**Vehicle**	3.19 ± 0.28	0.78 ± 0.02	0.76 ± 0.04	0.44 ± 0.03	0.53 ± 0.03
**4 mg/kg**	3.35 ± 0.31	0.81 ± 0.01	0.80 ± 0.04	0.43 ± 0.02	0.51 ± 0.03
**8 mg/kg**	3.27 ± 0.39	0.78 ± 0.02	0.73 ± 0.03	0.47 ± 0.03	0.54 ± 0.02
**16 mg/kg**	2.64 ± 0.37	0.76 ± 0.03	0.79 ± 0.04	0.43 ± 0.06	0.51 ± 0.04
**32 mg/kg**	2.93 ± 0.41	0.73 ± 0.03	0.80 ± 0.03	0.49 ± 0.05	0.46 ± 0.04
**64 mg/kg**	1.76 ± 0.27	0.67 ± 0.05	0.76 ± 0.05	0.50 ± 0.06	0.46 ± 0.06

Note: NTLS = Non-Target Lose Shift; NTWS = Non-Target Win-Stay; TLS = Target Lose-Shift; TWS = Target Win-Stay

Supplementary Figures

Supplementary Figure 1. Cross-species PRL Task Design

The human (A) and rat (B) versions of the PRL task were designed to be procedurally similar. During each trial, two stimuli were presented simultaneously and subjects used a keyboard (humans) or levers (rats) to select one of the stimuli. One stimulus was randomly selected to be the target at the beginning of each test session. Selection of the target was rewarded with 80% probability, whereas selection of the non-target stimulus was rewarded with 20% probability. After each response, a high or low frequency tone signaled the subsequent presentation or omission of a reward (counterbalanced). When the target was selected on 8 consecutive trials, the reinforcement contingencies reversed such that the other stimulus became the target. Each test session lasted 300 trials for both species, and the total number of reversals and other measures (e.g., target win-stay and lose-shift probabilities) were recorded.

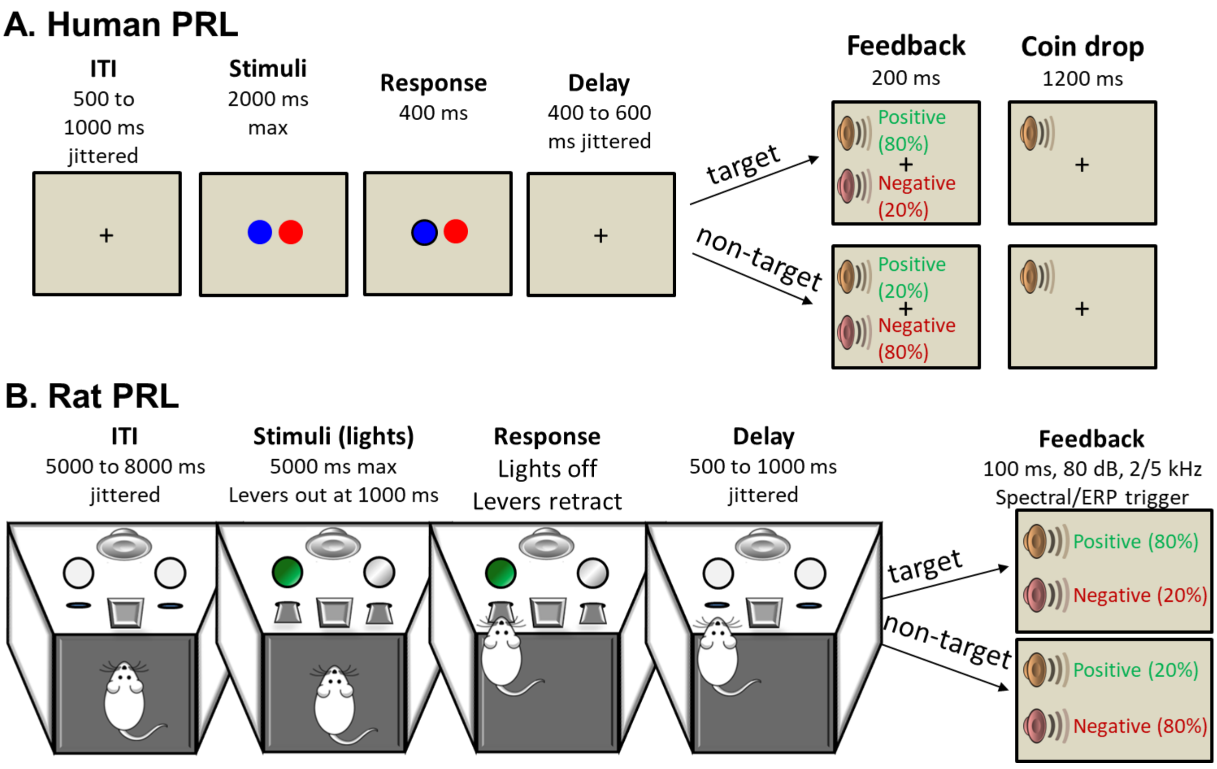

Supplementary Figure 2. Simulated vs. Actual PRL Behavioral Data

To confirm the validity of the selected Q-learning model we first performed a posterior predictive check by simulating PRL performance (n = 30) using the parameter values obtained from fitting the model to behavior. There was a close correspondence between the actual and simulated PRL performance for the number of completed reversals (A), target win-stay (B), and target lose-shift responses (C). Additionally, we ensured that the three model parameters of the chosen model (alpha, beta, forget parameters) were accurately estimated. We simulated PRL performance using known values for each parameter and then fitted the Q-learning model to the simulated PRL data. A positive correlation for each of the three model parameters was evident between the simulated and fitted values (D – F), indicating that the parameters were recoverable and accurately estimated.

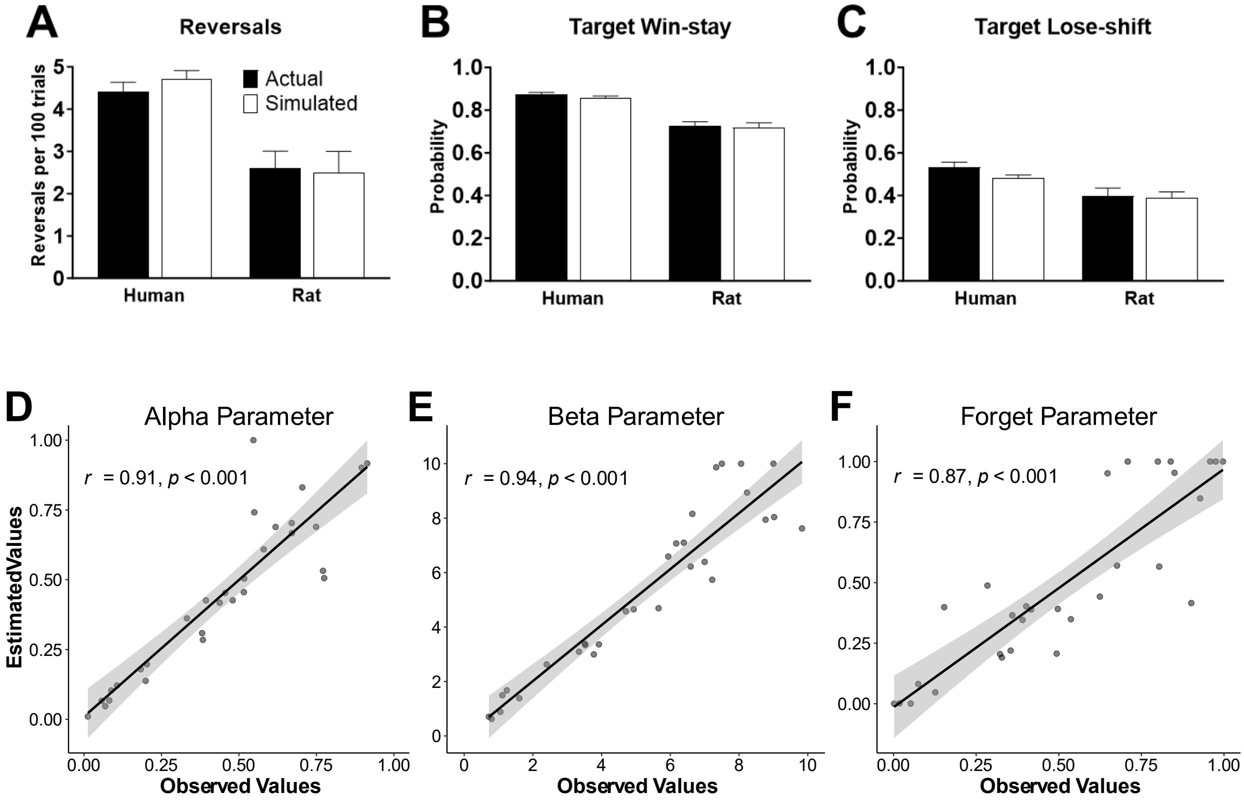

Supplemental Figure 3. Q and PE Values During PRL Task Performance

Representative Q and PE values are displayed across a test session for humans (A) and rats (B). Blue and orange lines represent Q and PE values, respectively, across trials. The dots above and below Q and PE values represent responses on the left/right apertures. The dotted lines represent periods during which the response criterion is achieved and a reversal is recorded. Trial number is indicated along the x-axis. Across an entire test session, Q values were significantly greater for target vs. non-target stimuli in both humans (*n* = 54) and rats (*n* = 11) (C).

A. Representative Human Q and PE values

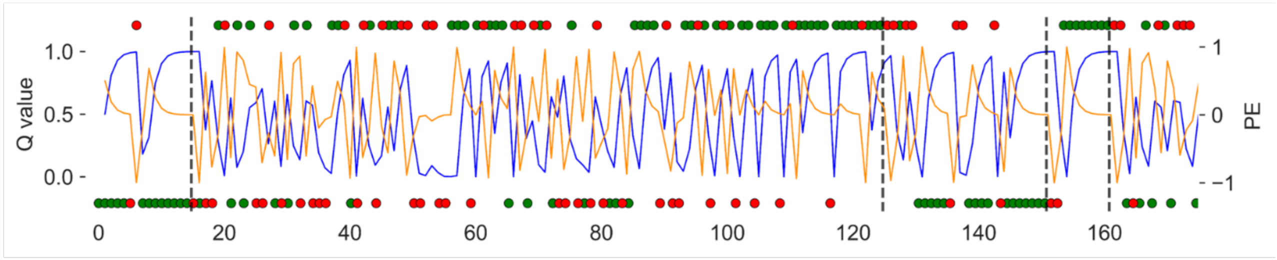

B. Representative Rat Q and PE values

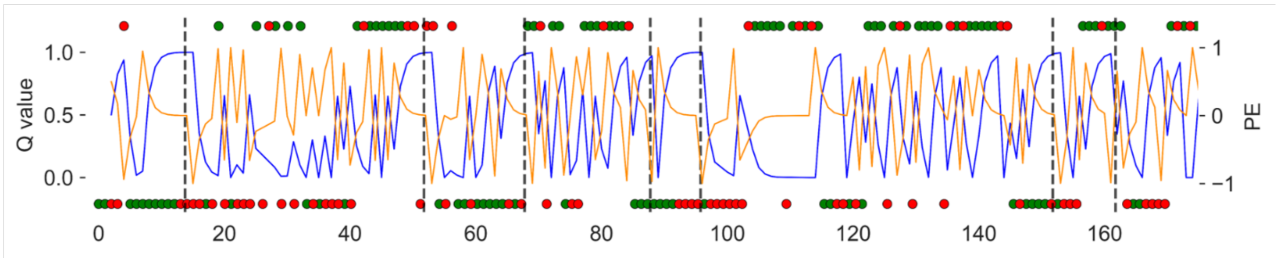

C. Average Q values for target and non-target stimuli

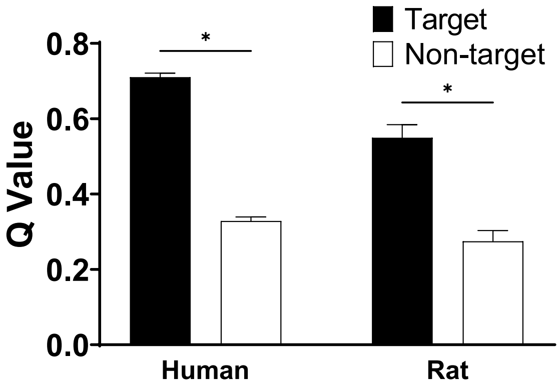

Supplemental Figure 4. Q-learning Model Parameter Association with Reversals

Alpha (A, B) and forget (C, D) parameters were correlated with number of reversals per 100 trials in both humans (n = 54) and rats (n = 11) using simple linear regression. None of these model parameters were significantly associated with reversals in either species (all r values < 0.55; all p values > 0.05).

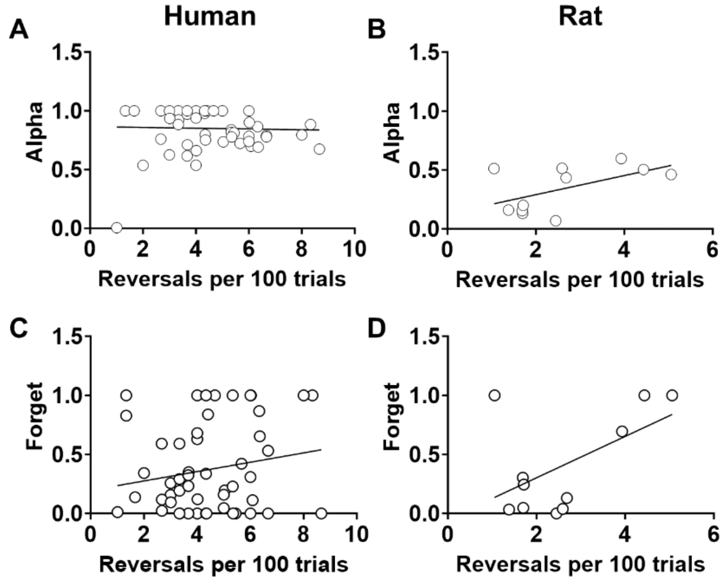

Supplemental Figure 5. Relationship between neural activity and the outcome and expected value components of the PE

The regression model presented in Figure 3 of the main text revealed a positive relationship between neural activity and reward PEs. However, the sign of reward PEs is dependent on the outcome received. Therefore, to confirm that this relationship was due to prediction error signaling and not simply due to the outcome valence, it was important to disentangle this potential collinearity by performing a secondary regression analysis that included outcome and expected value (i.e., the two sub-components of a reward prediction error) as model predictors. As expected, a positive regression coefficient was evident in humans (A) and rodents (B) for the response outcome (blue line) and a negative relationship was evident for expected value (red line). Importantly, this divergence coincided with the timepoint in the first model where reward PEs was positively associated with neural activity. Thus, this relationship is likely due to an association with prediction error signaling and not simply due to outcome valence.

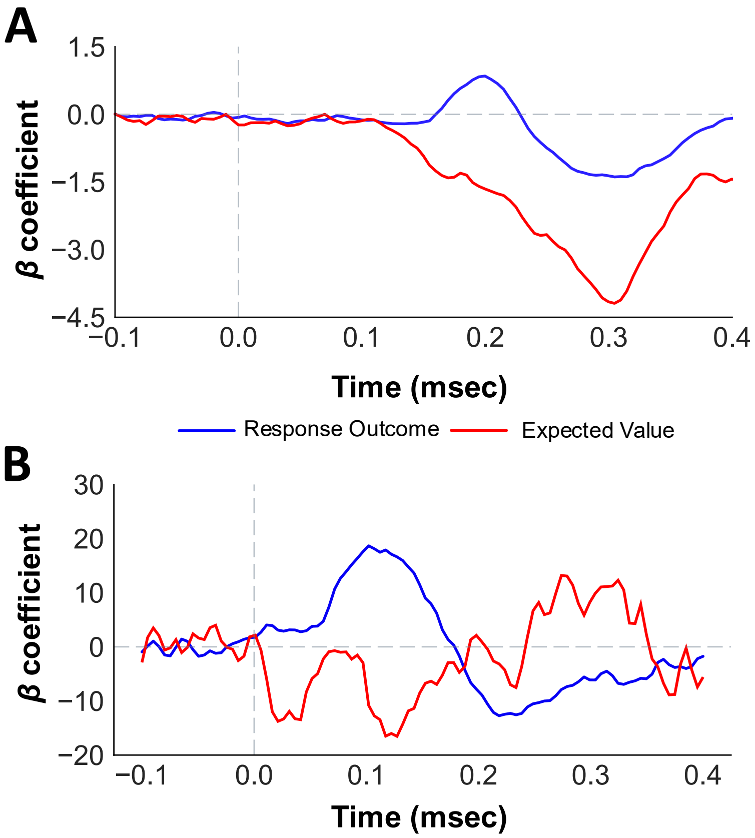

Supplemental Figure 6. Feedback locked ERP (human) and LFP (rodent) waveforms for each administered dose of modafinil

Human (n = 30) and rodent (n = 11) subjects each completed the PRL task after being administered different doses of modafinil and EEG data were captured throughout these sessions. Human participants were administered 100 mg (A) and 200 mg (B) doses, while rodents were administered weight-based doses of 4mg (C), 8mg (D), 16 mg (E), 32 mg (F), and 64 mg (G) per kilogram of body weight. All plots present the evoked neural activity following the delivery of feedback for the various trial types of the task (rewarded target = blue solid line; nonrewarded target = red solid line; rewarded non-target = blue dotted line; non-rewarded non-target = red dotted line) and expectancy-based difference waveforms (Expected = Rewarded Target – Non-rewarded Non-Target, and Unexpected = Rewarded Non-Target – Non-rewarded Target) and identify a Reward Positivity (RewP) or RewP-like signal in humans and rats, respectively.

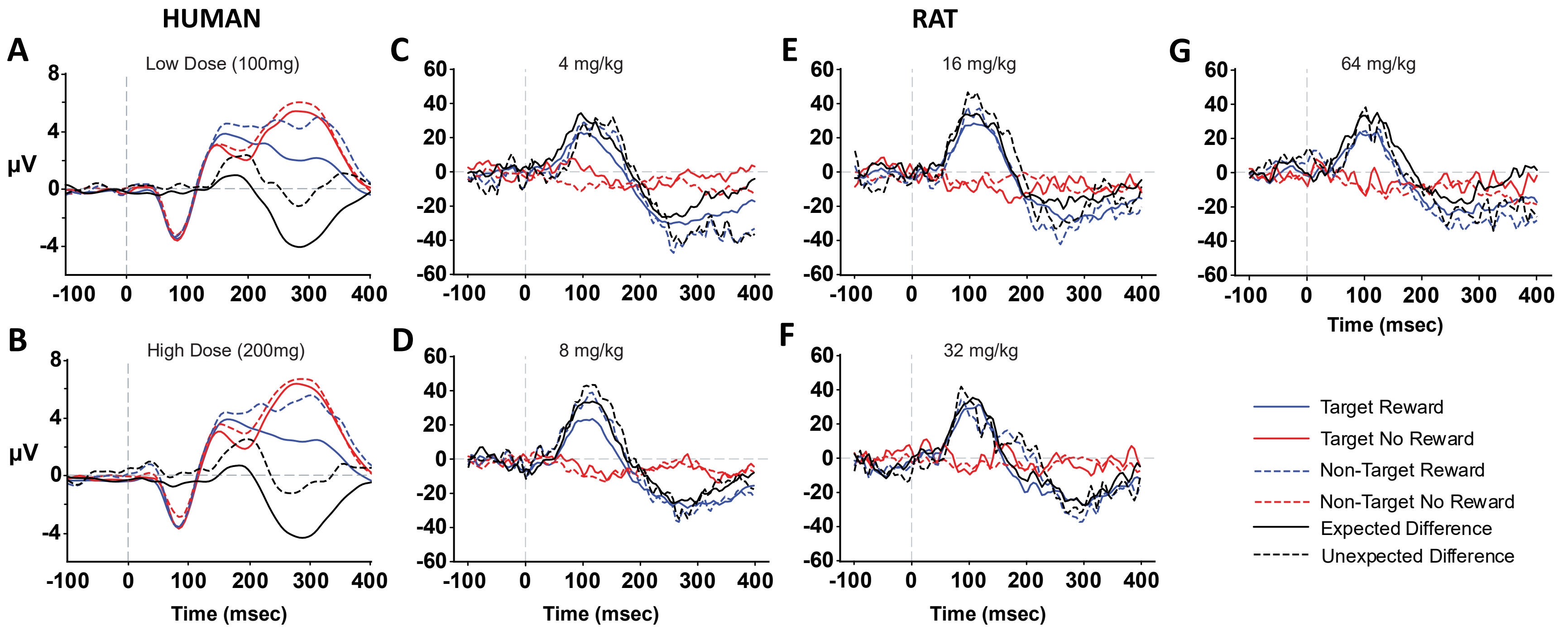

Supplementary References

1. Schlagenhauf F. et al. Striatal dysfunction during reversal learning in unmedicated schizophrenia patients. *NeuroImage* **89**, 171-80 (2014).
2. Katahira K. The relation between reinforcement learning parameters and the influence of reinforcement history on choice behavior. *Journal of mathematical psychology* **66**, 59-69 (2015).
3. Hattori R., Danskin B., Babic Z., Mlynaryk N. & Komiyama T. Area-specificity and plasticity of history-dependent value coding during learning. *Cell* **177(7)**, 1858-72.e15 (2019).
4. Weinstein A., El-Deredy W., Chabert S., Fuentes M. editors. Fitting human decision making models using python. *Proceedings of the 15th Python in Science Conference*. Ed. by Sebastian Benthall and Scott Rostrup, 1-6 (2016).
5. Wilson R.C. & Collins A.G.E. Ten simple rules for the computational modeling of behavioral data. *eLife* **8**:e49547 (2019).
6. Zhang L. & Gläscher J. A brain network supporting social influences in human decision-making. *Science Advances* **6**:eabb4159 (2020).
7. Barnes S.A., Dillon D.G., Young J.W., Thomas M.L., Faget L., Yoo J.H., Der-Avakian A., Hnasko T.S., Geyer M.A. & Ramanathan D.S. Modulation of ventromedial orbitofrontal cortical glutamatergic activity affects the explore-exploit balance and influences value-based decision-making. *Cerebral Cortex* **33(10)**, 5783-5796 (2023)
