# Supplementary material for: Identification of conserved frontal neurophysiological markers of cognitive flexibility in humans and rats
Source: Commun Biol. 2025 Aug 23;8:1268. doi: 10.1038/s42003-025-08729-x (PMC12375130; doi:10.1038/s42003-025-08729-x)
Supplement: Supplementary file 3 — Reporting Summary [file 42003_2025_8729_MOESM3_ESM.pdf]

nature portfolio

Corresponding author(s): Diego A. Pizzagalli

Last updated by author(s): Aug 7, 2025

Reporting Summary

Nature Portfolio wishes to improve the reproducibility of the work that we publish. This form provides structure for consistency and transparency in reporting. For further information on Nature Portfolio policies, see our Editorial Policies and the Editorial Policy Checklist.

Statistics

For all statistical analyses, confirm that the following items are present in the figure legend, table legend, main text, or Methods section.

n/a	Confirmed	
☐	☒	The exact sample size (*n*) for each experimental group/condition, given as a discrete number and unit of measurement
☐	☒	A statement on whether measurements were taken from distinct samples or whether the same sample was measured repeatedly
☐	☒	The statistical test(s) used AND whether they are one- or two-sided<br>*Only common tests should be described solely by name; describe more complex techniques in the Methods section.*
☐	☒	A description of all covariates tested
☐	☒	A description of any assumptions or corrections, such as tests of normality and adjustment for multiple comparisons
☐	☒	A full description of the statistical parameters including central tendency (e.g. means) or other basic estimates (e.g. regression coefficient) AND variation (e.g. standard deviation) or associated estimates of uncertainty (e.g. confidence intervals)
☐	☒	For null hypothesis testing, the test statistic (e.g. *F*, *t*, *r*) with confidence intervals, effect sizes, degrees of freedom and *P* value noted<br>*Give P values as exact values whenever suitable.*
☒	☐	For Bayesian analysis, information on the choice of priors and Markov chain Monte Carlo settings
☒	☐	For hierarchical and complex designs, identification of the appropriate level for tests and full reporting of outcomes
☐	☒	Estimates of effect sizes (e.g. Cohen's *d*, Pearson's *r*), indicating how they were calculated

Our web collection on statistics for biologists contains articles on many of the points above.

Software and code

Policy information about availability of computer code

Data collection	Human EEG data were collected using BrainVision recorder; Rodent EEG data were collected using MED-PC V software.
Data analysis	The following software were used in preprocessing raw data and subsequent analysis: BrainVision Analzyer (v2.0), Python (v3.7.1), NumPy Python library (v1.21.5), pandas Python Library (v1.1.5), SciPy Python Library (v1.4.1), matplotlib Python library (v3.5.3), statsmodels Python Library (v0.13.5), IBM SPSS Statistics (v24), and GraphPad Prism (v8).

For manuscripts utilizing custom algorithms or software that are central to the research but not yet described in published literature, software must be made available to editors and reviewers. We strongly encourage code deposition in a community repository (e.g. GitHub). See the Nature Portfolio guidelines for submitting code & software for further information.

Data

Policy information about availability of data

All manuscripts must include a data availability statement. This statement should provide the following information, where applicable:

- Accession codes, unique identifiers, or web links for publicly available datasets
- A description of any restrictions on data availability
- For clinical datasets or third party data, please ensure that the statement adheres to our policy

Numerical source data used in the present manuscript are hosted on OSF and available at: osf.io/gxkdv. All other data (i.e., raw EEG recordings) that support the findings of this study are available from the corresponding author upon reasonable request.

Research involving human participants, their data, or biological material

Policy information about studies with human participants or human data. See also policy information about sex, gender (identity/presentation), and sexual orientation and race, ethnicity and racism.

Reporting on sex and gender	Participants were asked to report on their Sex as either Male or Female. We did not examine the effect of Sex in the present work as we had no specific hypotheses regarding its effect relevant to the manuscripts aim of evaluating neurophysiological signatures of reward across species.
Reporting on race, ethnicity, or other socially relevant groupings	We have not reported or used any socially constructed or socially relevant categorization variables in the present work as we had no specific hypotheses regarding their effect relevant to the manuscripts main aim of evaluating neurophysiological signatures of reward across species.
Population characteristics	Population characteristics are provided in the Methods section of the manuscript.
Recruitment	Participants were recruited from the Greater Boston Community using a combination of locally placed advertisements, Online and Social Media advertisements, as well as Mass General Brigham's Rally collaborative research platform.
Ethics oversight	All human study procedures were approved by the Mass General Brigham Institutional Review Board, and subjects provided written informed consent in the presence of a medical doctor prior to participation.

Note that full information on the approval of the study protocol must also be provided in the manuscript.

Field-specific reporting

Please select the one below that is the best fit for your research. If you are not sure, read the appropriate sections before making your selection.

☒ Life sciences ☐ Behavioural & social sciences ☐ Ecological, evolutionary & environmental sciences

For a reference copy of the document with all sections, see nature.com/documents/nr-reporting-summary-flat.pdf

Life sciences study design

All studies must disclose on these points even when the disclosure is negative.

Sample size	Two cohorts of human participants, aged 18-45 years, were recruited for the present work. From Cohort 1 (n = 63) a total of n = 54 individuals (19 male, 35 female) were retained for analysis, and from Cohort 2 (n = 30) a total of n = 29 individuals (14 male, 15 female) were retained for analysis.
Data exclusions	EEG data from n = 20 individuals from Cohort 1 and n = 1 individual from Cohort 2 were excluded prior to analysis due to poor signal quality.
Replication	For the human portion of the study, Cohort 1 and Cohort 2 each completed the PRL task in a non-manipulated session (i.e., the only study session for Cohort 1, and the Placebo condition for Cohort 2) which provides a direct internal replication of the task and associated neural responses. In addition, for both species the within-subjects design of the Modafinil aspect of the study also provides some degree of replication. It is, however, worth noting that there was no independent replication of the Modafinil study.
Randomization	Cohort 1 only completed a single study session that did not involve any intervention or experimental manipulation, and so there was no randomization required. Cohort 2 completed three study sessions at which they were administered one of 3 doses of Modafinil. Sessions were separated by at least one week and randomised using a double-blind, within-subjects, placebo-controlled design.
Blinding	Cohort 1 completed a single study session that did not involve any intervention or experimental manipulation and so there was no need for blinding. The three study sessions for Cohort 2 were double-blinded, and investigator blinding was maintained until after the initial analysis was completed.

Reporting for specific materials, systems and methods

We require information from authors about some types of materials, experimental systems and methods used in many studies. Here, indicate whether each material, system or method listed is relevant to your study. If you are not sure if a list item applies to your research, read the appropriate section before selecting a response.

Materials & experimental systems

n/a	Involved in the study
☒	☐ Antibodies
☒	☐ Eukaryotic cell lines
☒	☐ Palaeontology and archaeology
☐	☒ Animals and other organisms
☐	☒ Clinical data
☒	☐ Dual use research of concern
☒	☐ Plants

Methods

n/a	Involved in the study
☒	☐ ChIP-seq
☒	☐ Flow cytometry
☒	☐ MRI-based neuroimaging

Animals and other research organisms

Policy information about studies involving animals; ARRIVE guidelines recommended for reporting animal research, and Sex and Gender in Research

Laboratory animals	Rodent behavioral and EEG data were collected from n = 11 adult Wistar rats.
Wild animals	This study did not involve wild animals.
Reporting on sex	Of the n = 11 animals used, n = 5 were male, n = 6 were female. As with our human analysis, we did not examine the effect of subject Sex in the present work as we had no specific hypotheses regarding its effect relevant to the manuscripts aim of evaluating neurophysiological signatures of reward across species.
Field-collected samples	This study did not involve samples collected from the field.
Ethics oversight	All rodent study procedures were conducted in accordance with guidelines from the National Institute of Health and the Association for Assessment and Accreditation of Laboratory Animal Care, and approved by the UC San Diego Institutional Animal Care and Use Committee.

Note that full information on the approval of the study protocol must also be provided in the manuscript.

Clinical data

Policy information about clinical studies
All manuscripts should comply with the ICMJE guidelines for publication of clinical research and a completed CONSORT checklist must be included with all submissions.

Clinical trial registration	NCT02855229
Study protocol	The protocols relevant to the present work are described in the Methods section of the manuscript. More detailed descriptions of the full protocols can be found at the ClinicTrials.gov listing (NCT02855229)
Data collection	Human EEG data were collected in an electronically and acoustically shielded EEG booth housed at McLean Hospital in Belmont Massachusetts, USA. Data from Cohort 1 were collected between April 2017 and October 2017, and the data from Cohort 2 were collected between November 2018 to October 2019.<br><br>Rodent EEG data were collected in a Plexiglas operant condition box enclosed in a Faraday cage that was housed at the University of California, San Diego in La Jolla California, USA. All rodent data were collected between October 2018 and January 2020.
Outcomes	The primary outcome of interest was the Reward Positivity (AKA the feedback-related negativity) event-related component. This component was derived from individual electroencephalography recordings and quantified as the average amplitude between 165 and 225ms post-feedback for humans and 60-160ms post-feedback for rodents; a complete description of the generation of this component is provided in the Methods section of the manuscript. Other outcomes parameters of interest include the Alpha, Beta, and Forget parameters that were derived from fitting a Q-Learning model to human and rodent task behavior separately. A detailed description of this model and the iterative process used to determine its structure is provided in the Supplementary materials.

Plants

Seed stocks

Report on the source of all seed stocks or other plant material used. If applicable, state the seed stock centre and catalogue number. If plant specimens were collected from the field, describe the collection location, date and sampling procedures.

Novel plant genotypes

Describe the methods by which all novel plant genotypes were produced. This includes those generated by transgenic approaches, gene editing, chemical/radiation-based mutagenesis and hybridization. For transgenic lines, describe the transformation method, the number of independent lines analyzed and the generation upon which experiments were performed. For gene-edited lines, describe the editor used, the endogenous sequence targeted for editing, the targeting guide RNA sequence (if applicable) and how the editor was applied.

Authentication

Describe any authentication procedures for each seed stock used or novel genotype generated. Describe any experiments used to assess the effect of a mutation and, where applicable, how potential secondary effects (e.g. second site T-DNA insertions, mosiacism, off-target gene editing) were examined.
