# Supplementary material for: Identification of conserved frontal neurophysiological markers of cognitive flexibility in humans and rats
Source: Commun Biol. 2025 Aug 23;8:1268. doi: 10.1038/s42003-025-08729-x (PMC12375130; doi:10.1038/s42003-025-08729-x)
Supplement: Supplementary file 4 — Python Code for Human Data Analysis [file 42003_2025_8729_MOESM4_ESM.html]

Final Human Analysis


In [ ]:

```
import os
import pandas as pd
import numpy as np
import seaborn as sns
import matplotlib.pyplot as plt
import glob as glob
import scipy as sp
import matplotlib.pyplot as plt
import getpass
import math
import re
import warnings

plt.rcParams.update({'font.size': 16})
sns.set_style('darkgrid')
sns.color_palette('deep')
warnings.filterwarnings('ignore')
```

In [ ]:

```
filePath = "C:/Users/samba/Dropbox/Work Folder/Python Scripts/PRL/UH3_Project/Human_UH2/Analysis/" #Change the file path here to the location of the data
all_files = glob.glob(filePath + "/*.csv")

save_path = 'C:/Users/samba/Dropbox/Work Folder/Python Scripts/PRL/UH3_Project/Human/Processed_Data/'

# import all csv files in target dir and concat to df
file = []
for filename in all_files:
    df = pd.read_csv(filename, index_col=None, header=0)
    df['filename'] = filename
    file.append(df)
df = pd.concat(file, axis=0, ignore_index=True)
```

In [ ]:

```
data = df
```

In [ ]:

```
data['ERP_win'] = data.loc[:, 'T165':'T225'].mean(axis=1)
```

In [ ]:

```
def makePlots(input_data1, input_data2, input_data3, input_data4, title, measure1, measure2, measure3, measure4, fig_name):
    
    plt.rcParams.update({'font.size': 20})
    
    dataset1 = input_data1
    dataset2 = input_data2
    dataset3 = input_data3
    dataset4 = input_data4
    
    rat_plot1 = []
    for subj in dataset1.subject.unique(): 
        sdf1 = dataset1[(dataset1.subject == subj)]
        subj_id = pd.DataFrame(sdf1.subject.unique())    # grab rat ID
        p1 = sdf1.loc[:,'TNeg100':'T545'].mean(axis=0)  # column averages for each rat and each measure
        plot1 = pd.DataFrame(p1).T       
        plot1_and_ID = pd.concat([subj_id, plot1], axis=1)
        rat_plot1.append(plot1_and_ID)

    TR_plot = pd.concat(rat_plot1)
    TR_plot.reset_index(inplace=True,drop=True)
    
    rat_plot2 = []
    for subj in dataset2.subject.unique(): 
        sdf2 = dataset2[(dataset2.subject == subj)]
        subj_id = pd.DataFrame(sdf2.subject.unique())    # grab rat ID
        p2 = sdf2.loc[:,'TNeg100':'T545'].mean(axis=0)  # column averages for each rat and each measure
        plot2 = pd.DataFrame(p2).T  
        plot2_and_ID = pd.concat([subj_id, plot2], axis=1)
        rat_plot2.append(plot2_and_ID)

    TNR_plot = pd.concat(rat_plot2)
    TNR_plot.reset_index(inplace=True,drop=True)
  
    rat_plot3 = []
    for subj in dataset3.subject.unique(): 
        sdf3 = dataset3[(dataset3.subject == subj)]
        subj_id = pd.DataFrame(sdf3.subject.unique())    # grab rat ID
        p3 = sdf3.loc[:,'TNeg100':'T545'].mean(axis=0)  # column averages for each rat and each measure
        plot3 = pd.DataFrame(p3).T  
        plot3_and_ID = pd.concat([subj_id, plot3], axis=1)
        rat_plot3.append(plot3_and_ID)

    NTNR_plot = pd.concat(rat_plot3)
    NTNR_plot.reset_index(inplace=True,drop=True)

    rat_plot4 = []
    for subj in dataset4.subject.unique(): 
        sdf4 = dataset4[(dataset4.subject == subj)]
        subj_id = pd.DataFrame(sdf4.subject.unique())    # grab rat ID
        p4 = sdf4.loc[:,'TNeg100':'T545'].mean(axis=0)  # column averages for each rat and each measure
        plot4 = pd.DataFrame(p4).T       
        plot4_and_ID = pd.concat([subj_id, plot4], axis=1)
        rat_plot4.append(plot4_and_ID)

    NTR_plot = pd.concat(rat_plot4)
    NTR_plot.reset_index(inplace=True,drop=True)
    
    TR_error = np.std(TR_plot.loc[:, 'TNeg100':'T545'],axis=0)/np.sqrt(len(TR_plot)) 
    TNR_error = np.std(TNR_plot.loc[:, 'TNeg100':'T545'],axis=0)/np.sqrt(len(TNR_plot))     
    NTNR_error = np.std(NTNR_plot.loc[:, 'TNeg100':'T545'],axis=0)/np.sqrt(len(NTNR_plot)) 
    NTR_error = np.std(NTR_plot.loc[:, 'TNeg100':'T545'],axis=0)/np.sqrt(len(NTR_plot))     
    
    T_diff = np.subtract(TR_plot.loc[:, 'TNeg100':'T545'],TNR_plot.loc[:, 'TNeg100':'T545'])
    NT_diff = np.subtract(NTR_plot.loc[:, 'TNeg100':'T545'],NTNR_plot.loc[:, 'TNeg100':'T545'])
    T_diff_error = np.std(T_diff.loc[:, 'TNeg100':'T545'],axis=0)/np.sqrt(len(T_diff))   
    NT_diff_error = np.std(NT_diff.loc[:, 'TNeg100':'T545'],axis=0)/np.sqrt(len(NT_diff))   
    
    axis1 = TR_plot.columns[1:]
    
    bins = len(np.mean(TR_plot.loc[:, 'TNeg100':'T545'],axis=0))
    axis1 = np.linspace(-0.1, 0.545, num=bins)

    fig = plt.figure(figsize=(8, 12), dpi=600)
    ax1 = fig.add_subplot(311)

    ax1.set_facecolor('white')
    ax1.spines["top"].set_visible(False)
    ax1.spines["right"].set_visible(False)
    ax1.spines['left'].set_color('black')
    ax1.spines['bottom'].set_color('black')
    ax1.spines['left'].set_linewidth(1)
    ax1.spines['bottom'].set_linewidth(1)

    ax1.set_title(title)
    p1, = ax1.plot(axis1, np.mean(TR_plot.loc[:, 'TNeg100':'T545'],axis=0), linewidth=2, color='blue', label=measure1)
    p2, = ax1.plot(axis1, np.mean(TNR_plot.loc[:, 'TNeg100':'T545'],axis=0), linewidth=2, color='red', label=measure2)
    p3, = ax1.plot(axis1, np.mean(NTNR_plot.loc[:, 'TNeg100':'T545'],axis=0), linewidth=2, linestyle='--', color='red', label=measure3)
    p4, = ax1.plot(axis1, np.mean(NTR_plot.loc[:, 'TNeg100':'T545'],axis=0), linewidth=2, linestyle='--', color='blue', label=measure4)

    ax1.axvline(x=0, linewidth=3, linestyle='--', color='slategray', alpha=0.5)   
    ax1.axhline(y=0, linewidth=3, linestyle='--', color='slategray', alpha=0.5)   
    ax1.set_ylabel('activity (uV)')
    ax1.legend(handles=[p1, p2, p3, p4], loc='upper right')
    ax1.set_ylim(-0.000005, 0.000008)  
      
    ### plot 2 ###
    ax2 = fig.add_subplot(312)       
    ax2.set_facecolor('white')
    ax2.spines["top"].set_visible(False)
    ax2.spines["right"].set_visible(False)

    ax2.spines['left'].set_color('black')
    ax2.spines['bottom'].set_color('black')
    ax2.spines['left'].set_linewidth(1)
    ax2.spines['bottom'].set_linewidth(1)
    
    axis2 = axis1
    
    p1, = ax3.plot(axis2, np.mean(T_diff.loc[:, 'TNeg100':'T545'],axis=0), linewidth=2, color='black', label='T diff')
    p2, = ax3.plot(axis2, np.mean(NT_diff.loc[:, 'TNeg100':'T545'],axis=0), linewidth=2, linestyle='--', color='black', label='NT diff')
     
    ax2.axvline(x=0, linewidth=3, linestyle='--', color='slategray', alpha=0.5)   
    ax2.axhline(y=0, linewidth=3, linestyle='--', color='slategray', alpha=0.5)     
    ax2.set_ylabel('activity (uV)')
    ax2.legend(handles=[p1, p2], loc='upper right')
    ax2.set_xlabel('time surrounding tone (s)')
    ax2.set_ylim(-0.000005, 0.000008)    
             
    xticks = np.arange(-0.1, 0.51, 0.1)  # from -0.1 to 0.5 in 0.1 increments
    ax1.set_xticks(xticks)
    ax2.set_xticks(xticks)

    yticks = np.arange(-0.000006, 0.0000081, 0.000002)  # 1e-6 steps
    ax1.set_yticks(yticks)
    ax2.set_yticks(yticks)
    
    ax1.set_xlim(-0.1, 0.4)
    ax2.set_xlim(-0.1, 0.4)
    
    plt.tight_layout()
       
    #plt.savefig(save_path + fig_name + '.svg', transparent=False)
    
    return fig
```

In [ ]:

```
group = data

plot_data = group

plot_fig = makePlots(plot_data[(plot_data.reward == 1)&(plot_data.response == 'rich')], 
                 plot_data[(plot_data.reward == 0)&(plot_data.response == 'rich')],
                 plot_data[(plot_data.reward == 0)&(plot_data.response == 'lean')], 
                 plot_data[(plot_data.reward == 1)&(plot_data.response == 'lean')], 'Fcz', 'TR', 'TNR', 'NTNR', 'NTR', 'Fig 2 - human plot')
```

In [ ]:

```
def GLM_one_predictor(input_data, predictors):
        
    import statsmodels.api as sm
    import statsmodels.formula.api as smf

    activity = input_data.loc[:, 'TNeg100':'T545']

    intercept = []
    coef1 = []
    pvalues1 = []
    tvalues1 = []
    std1 = []

    for g in activity:

        timebin = activity[g]
        input_data['bin'] = timebin

        md = smf.glm("bin ~ PE", input_data, family=sm.families.Gaussian())
        mdf = md.fit()

        intercept.append(mdf.params['Intercept'])
        coef1.append(mdf.params[predictors[0]])
        pvalues1.append(mdf.pvalues[predictors[0]])
        tvalues1.append(mdf.tvalues[predictors[0]])
        std1.append(mdf.bse[predictors[0]])
      
    return intercept, coef1, std1, pvalues1
```

In [ ]:

```
def plot_one_betas(betas, pvals_corr, plot_pvals, adjust):
    
    betas = np.array(betas)
    pvals_corr = np.array(pvals_corr)
    
    bins = len(betas)

    axis1 = np.linspace(-0.1,0.545, num=bins)
   
    # Plot data
    import matplotlib.ticker as ticker
    parameters = {'axes.labelsize': 25, 'axes.titlesize': 35, 'xtick.labelsize': 20, 'ytick.labelsize': 20}
    plt.rcParams.update(parameters)
    
    fig = plt.figure(figsize=(8, 12), dpi=600)
    ax0 = fig.add_subplot(311)

    ax0.set_facecolor('white')

    ax0.spines["top"].set_visible(False)
    ax0.spines["right"].set_visible(False)

    ax0.spines['left'].set_color('black')
    ax0.spines['bottom'].set_color('black')
    ax0.spines['left'].set_linewidth(1)
    ax0.spines['bottom'].set_linewidth(1)

    p1, = ax0.plot(axis1, betas, linewidth=2, color='blue')

    if plot_pvals == 'yes':
        
        n_pvals = pvals_corr.shape
        max_y1 = betas.max() + adjust

        for i in pvals_corr:
            t = np.linspace(-0.1,0.545, num=bins)
            t05 = t[pvals_corr < 0.05]
            ax0.plot(t05, np.ones(t05.shape)*max_y1, '.', color='b', markersize=6)
    else:
        pass    
        
    ax0.axvline(x=0, linewidth=3, linestyle='--', color='slategray', alpha=0.5)   
    ax0.axhline(y=0, linewidth=3, linestyle='--', color='slategray', alpha=0.5)   
    
    xticks = np.arange(-0.1, 0.51, 0.1)
    yticks = np.arange(-0.0000004, 0.0000021, 0.0000004)
    
    ax0.set_xticks(xticks)
    ax0.set_yticks(yticks)

    ax0.set_ylabel('regression coefficient')
    ax0.set_xlabel('time surrounding tone (s)')
    
    ax0.set_xlim(-0.1, 0.4)
    
    #plt.savefig(save_path + 'Human_Fig3A.svg', transparent=False)
    
    plt.tight_layout()             
    
    
def predictActivity (PE, model_coefficient, model_intercept):
    
    """
    # function takes a PE value, the model coefficient and the intercept 
    value and returns the predicted GCaMP signal
    # https://datagy.io/python-sklearn-linear-regression/
    """   
    return (PE * model_coefficient) + model_intercept
```

In [ ]:

```
UH2_regression = data
UH2_regression.reward.replace({1:1, 0:-1},inplace=True)

PE_intercept, PE_coef, PE_sem, PE_pvalues = GLM_one_predictor(UH2_regression, predictors=['PE'])

#UH2_regression.to_csv(save_path + 'Fig3_Humna_Regression_Data.csv')
```

In [ ]:

```
plot_fig = plot_one_betas(PE_coef,
               PE_pvalues,
               plot_pvals='no', 
               adjust=10)
```

In [ ]:

```
predicted_60 = []
predicted_30 = []
predicted_0 = []
predicted_minus30 = []
predicted_minus60 = []

for x, y in zip(PE_coef, PE_intercept):
                predicted_60.append(predictActivity(0.6, x, y))
        
for x, y in zip(PE_coef, PE_intercept):
                predicted_30.append(predictActivity(0.3, x, y))
        
for x, y in zip(PE_coef, PE_intercept):
                predicted_0.append(predictActivity(0, x, y))
        
for x, y in zip(PE_coef, PE_intercept):
                predicted_minus30.append(predictActivity(-0.3, x, y))
        
for x, y in zip(PE_coef, PE_intercept):
                predicted_minus60.append(predictActivity(-0.6, x, y))
```

In [ ]:

```
bins = len(PE_coef)
axis1 = np.linspace(-0.1,0.545, num=bins)


fig = plt.figure(figsize=(8, 12), dpi=600)
ax0 = fig.add_subplot(311)

ax0.set_facecolor('white')

ax0.spines["top"].set_visible(False)
ax0.spines["right"].set_visible(False)

ax0.spines['left'].set_color('black')
ax0.spines['bottom'].set_color('black')
ax0.spines['left'].set_linewidth(1)
ax0.spines['bottom'].set_linewidth(1)

p1, = ax0.plot(axis1, predicted_60, linewidth=2, color='blue', label='PE, 0.6')
p2, = ax0.plot(axis1, predicted_30, linewidth=2, color='blue', linestyle='--', label='PE, 0.3')

p4, = ax0.plot(axis1, predicted_minus30, linewidth=2, color='red', linestyle='--', label='PE, -0.3')
p5, = ax0.plot(axis1, predicted_minus60, linewidth=2, color='red', label='PE, -0.6')

ax0.axvline(x=0, linewidth=3, linestyle='--', color='slategray', alpha=0.5)   
ax0.axhline(y=0, linewidth=3, linestyle='--', color='slategray', alpha=0.5)   
ax0.legend(handles=[p1, p2, p4, p5], loc='upper right')

ax0.set_ylabel('Predicted Fzc activity')
ax0.set_xlabel('time surrounding tone (s)')

xticks = np.arange(-0.1, 0.51, 0.1)
yticks = np.arange(-0.000005, 0.0000051, 0.0000025)

ax0.set_xticks(xticks)
ax0.set_yticks(yticks)

ax0.set_xlim(-0.1, 0.4)

#plt.savefig(save_path + 'Human_Fig3C.svg', transparent=False)
    
plt.tight_layout()
```

In [ ]:

```
Hi_PE = data[(data.PE >0.5)]
Lo_PE = data[(data.PE <-0.5)]

axis1 = np.linspace(-0.1,0.545, num=bins)

fig = plt.figure(figsize=(8, 12), dpi=600)
ax0 = fig.add_subplot(311)

ax0.set_facecolor('white')

ax0.spines["top"].set_visible(False)
ax0.spines["right"].set_visible(False)

ax0.spines['left'].set_color('black')
ax0.spines['bottom'].set_color('black')
ax0.spines['left'].set_linewidth(1)
ax0.spines['bottom'].set_linewidth(1)


p1, = ax0.plot(axis1, np.mean(Hi_PE.loc[:, 'TNeg100':'T545'],axis=0), linewidth=2, color='blue', label='PE >0.5')
p2, = ax0.plot(axis1, np.mean(Lo_PE.loc[:, 'TNeg100':'T545'],axis=0), linewidth=2, color='red', label='PE <0.5')

ax0.axvline(x=0, linewidth=3, linestyle='--', color='slategray', alpha=0.5)   
ax0.axhline(y=0, linewidth=3, linestyle='--', color='slategray', alpha=0.5)   
ax0.legend(handles=[p1, p2], loc='upper right')

ax0.set_ylabel('activity (uV)')
ax0.set_xlabel('time surrounding tone (s)')

xticks = np.arange(-0.1, 0.51, 0.1)
yticks = np.arange(-0.000006, 0.0000091, 0.000003)

ax0.set_xticks(xticks)
ax0.set_yticks(yticks)

ax0.set_xlim(-0.1, 0.4)

#plt.savefig(save_path + 'Human_Fig3E.svg', transparent=False)
    
plt.tight_layout()
```

In [ ]:

```
def GLM_two_predictor(input_data, predictors):
        
    import statsmodels.api as sm
    import statsmodels.formula.api as smf

    activity = input_data.loc[:, 'TNeg100':'T545']

    intercept = []
    coef1 = []
    coef2 = []
    pvalues1 = []
    pvalues2 = []
    tvalues1 = []
    tvalues2 = []
    std1 = []
    std2 = []

    for g in activity:

        timebin = activity[g]
        input_data['bin'] = timebin

        md = smf.glm("bin ~ reward + Q_Chosen", input_data, family=sm.families.Gaussian())
        mdf = md.fit()

        intercept.append(mdf.params['Intercept'])
        coef1.append(mdf.params[predictors[0]])
        coef2.append(mdf.params[predictors[1]])
        pvalues1.append(mdf.pvalues[predictors[0]])
        pvalues2.append(mdf.pvalues[predictors[1]])
        tvalues1.append(mdf.tvalues[predictors[0]])
        tvalues2.append(mdf.tvalues[predictors[1]])
        std1.append(mdf.bse[predictors[0]])
        std2.append(mdf.bse[predictors[1]])
      
    return intercept, coef1, coef2, std1, std2, pvalues1, pvalues2


def plot_two_betas(betas1, betas2, pvals_corr1, pvals_corr2, plot_pvals, adjust):
    
    betas1 = np.array(betas1)
    betas2 = np.array(betas2)
    pvals_corr1 = np.array(pvals_corr1)
    pvals_corr2 = np.array(pvals_corr2)
    
    bins = len(betas1)

    axis1 = np.linspace(-0.1,0.545, num=bins)
   
    import matplotlib.ticker as ticker
    parameters = {'axes.labelsize': 25, 'axes.titlesize': 35, 'xtick.labelsize': 20, 'ytick.labelsize': 20}
    plt.rcParams.update(parameters)
    
    fig = plt.figure(figsize=(8, 12), dpi=600)
    ax0 = fig.add_subplot(311)

    ax0.set_facecolor('white')

    ax0.spines["top"].set_visible(False)
    ax0.spines["right"].set_visible(False)

    ax0.spines['left'].set_color('black')
    ax0.spines['bottom'].set_color('black')
    ax0.spines['left'].set_linewidth(1)
    ax0.spines['bottom'].set_linewidth(1)

    p1, = ax0.plot(axis1, betas1, linewidth=2, color='blue')
    p1, = ax0.plot(axis1, betas2, linewidth=2, color='red')

    if plot_pvals == 'yes':
        
        n_pvals = pvals_corr.shape
        max_y1 = betas.max() + adjust

        for i in pvals_corr:
            t = np.linspace(-0.1,0.545, num=bins)
            t05 = t[pvals_corr < 0.05]
            ax0.plot(t05, np.ones(t05.shape)*max_y1, '.', color='b', markersize=6)
    else:
        pass    
        
    ax0.axvline(x=0, linewidth=3, linestyle='--', color='slategray', alpha=0.5)   
    ax0.axhline(y=0, linewidth=3, linestyle='--', color='slategray', alpha=0.5)   
    
    xticks = np.arange(-0.1, 0.51, 0.1)
    yticks = np.arange(-0.0000045, 0.0000016, 0.0000015)
    
    ax0.set_xticks(xticks)
    ax0.set_yticks(yticks)

    ax0.set_ylabel('regression coefficient')
    ax0.set_xlabel('time surrounding tone (s)')
    
    ax0.set_xlim(-0.1, 0.4)
    
    #plt.savefig(save_path + 'Human_SuppFig5A.svg', transparent=False)
    
    plt.tight_layout()
```

In [ ]:

```
rew_val_intercept, rew_val_coef1, rew_val_coef2, rew_val_sem1, rew_val_sem2, rew_val_pvalues1, rew_val_pvalues2 = GLM_two_predictor(UH2_regression, predictors=['reward', 'Q_Chosen'])
```

In [ ]:

```
plot_fig = plot_two_betas(rew_val_coef1, rew_val_coef2,
                          rew_val_sem1, rew_val_sem2,
                          plot_pvals='no', 
                          adjust=10)
```

In [ ]:

```

```
