# Supplementary material for: Identification of conserved frontal neurophysiological markers of cognitive flexibility in humans and rats
Source: Commun Biol. 2025 Aug 23;8:1268. doi: 10.1038/s42003-025-08729-x (PMC12375130; doi:10.1038/s42003-025-08729-x)
Supplement: Supplementary file 5 — R Code for analyses and plots [file 42003_2025_8729_MOESM5_ESM.html]

UH3\_Modafinil - ANOVAs & Figures


# UH3\_Modafinil - ANOVAs & Figures

#### Ty Lees

#### 2025-08-08

# 0.0 Workspace Prep

```
knitr::opts_chunk$set(echo = TRUE)

#Load poackages/libraries
packs = list("ggplot2", "psych", "emmeans", "lme4", "nlme", "lmerTest", "Matrix", "effects", "effectsize", "rstatix", "tidyr", "dplyr", "reshape2", "afex", "readxl", "Hmisc", "RcmdrMisc", "stringr", "reghelper")
lapply(packs, require, character.only = T)
```

```
# Set Options
options(max.print=1000)
# Set this to ensure consistent results from lmerTest::anova() and summary()
## Makes contrasts orthogonal, specifically useful for the omnibus type 3 tests
options(contrasts = c("contr.sum","contr.poly"))
options(scipen = 999)
```

# 1.0 - ANOVA Models

## 1.1 - Figure 2 Models

### 1.1.1 - Set-up and data Import

```
# Load in data from Figure 2
## Human ERP
hum_erp_aovdat <- readxl::read_xlsx("Figure2_data.xlsx", sheet = 1)
hum_erp_aovdat$response <- factor(hum_erp_aovdat$response, levels = c("Nontarget", "Target"))
hum_erp_aovdat$reward <- factor(hum_erp_aovdat$reward, levels = c("Nonreward", "Reward"))
hum_erp_aovdat$trial_type <- factor(hum_erp_aovdat$trial_type, levels = c("NTNR", "NTR", "TR", "TNR"))

## Human Pe
hum_pe_aovdat <- readxl::read_xlsx("Figure2_data.xlsx", sheet = 2)
hum_pe_aovdat$response <- factor(hum_pe_aovdat$response, levels = c("Nontarget", "Target"))
hum_pe_aovdat$reward <- factor(hum_pe_aovdat$reward, levels = c("Nonreward", "Reward"))
hum_pe_aovdat$trial_type <- factor(hum_pe_aovdat$trial_type, levels = c("NTNR", "NTR", "TR", "TNR"))
  
## Rodent ERP
rat_erp_aovdat <- readxl::read_xlsx("Figure2_data.xlsx", sheet = 3)
rat_erp_aovdat$response <- factor(rat_erp_aovdat$response, levels = c("Nontarget", "Target"))
rat_erp_aovdat$reward <- factor(rat_erp_aovdat$reward, levels = c("Nonreward", "Reward"))
rat_erp_aovdat$trial_type <- factor(rat_erp_aovdat$trial_type, levels = c("NTNR", "NTR", "TR", "TNR"))

## Rodent PE
rat_pe_aovdat <- readxl::read_xlsx("Figure2_data.xlsx", sheet = 4)
rat_pe_aovdat$response <- factor(rat_pe_aovdat$response, levels = c("Nontarget", "Target"))
rat_pe_aovdat$reward <- factor(rat_pe_aovdat$reward, levels = c("Nonreward", "Reward"))
rat_pe_aovdat$trial_type <- factor(rat_pe_aovdat$trial_type, levels = c("NTNR", "NTR", "TR", "TNR"))

afex_options(correction_aov = "none")
```

### 1.1.2 - ERPs

```
# Human ERP 2-way
hum_erp_m1 <- aov_ez("subject", "ERP_win", hum_erp_aovdat, within = c("response", "reward")); hum_erp_m1
## Posthoc tests
em1 <- emmeans::emmeans(hum_erp_m1, ~ reward|response); em1
pairs(em1, adjust = "bonferroni")

hum_erp_tdat <- hum_erp_aovdat %>%
                  dplyr::filter(trial_type == "TR" | trial_type == "TNR")
pairwise.t.test(hum_erp_tdat$ERP_win, hum_erp_tdat$reward, p.adjust.method = "bonferroni", paired = TRUE)

# Human ERP 1-way
hum_erp_m2 <- aov_ez("subject", "ERP_win", hum_erp_aovdat, within = c("trial_type")); hum_erp_m2


# Rodent ERP 2-way
rod_erp_m1 <- aov_ez("subject", "ERP_win", rat_erp_aovdat, within = c("response", "reward")); rod_erp_m1
## Posthoc tests
em2 <- emmeans::emmeans(rod_erp_m1, ~ reward|response); em2
pairs(em2, adjust = "bonferroni")

rat_erp_tdat <- rat_erp_aovdat %>%
                  dplyr::filter(trial_type == "TR" | trial_type == "TNR")
pairwise.t.test(rat_erp_tdat$ERP_win, rat_erp_tdat$reward, p.adjust.method = "bonferroni", paired = TRUE)

# Rodent ERP 1-way
rod_erp_m2 <- aov_ez("subject", "ERP_win", rat_erp_aovdat, within = c("trial_type")); rod_erp_m2
```

### 1.1.3 - PE

```
# Human PE 2-way
hum_pe_m1 <- aov_ez("subject", "PE", hum_pe_aovdat, within = c("response", "reward")); hum_pe_m1
## Posthoc tests
em3 <- emmeans::emmeans(hum_pe_m1, ~ reward|response); em3
pairs(em3, adjust = "bonferroni")

hum_erp_ptdat <- hum_pe_aovdat %>%
                  dplyr::filter(trial_type == "TR" | trial_type == "TNR")
pairwise.t.test(hum_erp_ptdat$PE, hum_erp_ptdat$reward, p.adjust.method = "bonferroni", paired = TRUE)

# Human PE 1-way
hum_pe_m2 <- aov_ez("subject", "PE", hum_pe_aovdat, within = c("trial_type")); hum_pe_m2


# Rodent PE 2-way
rod_pe_m1 <- aov_ez("subject", "PE", rat_pe_aovdat, within = c("response", "reward")); rod_pe_m1
## Posthoc tests
em4 <- emmeans::emmeans(rod_pe_m1, ~ reward|response); em4
pairs(em4, adjust = "bonferroni")

rat_pe_tdat <- rat_pe_aovdat %>%
                  dplyr::filter(trial_type == "TR" | trial_type == "TNR")
pairwise.t.test(rat_pe_tdat$PE, rat_pe_tdat$reward, p.adjust.method = "bonferroni", paired = TRUE)

# Rodent PE 1-way
rod_pe_m2 <- aov_ez("subject", "PE", rat_pe_aovdat, within = c("trial_type")); rod_pe_m2
```

## 1.2 - Figure 4 Models

### 1.2.1 - Set-up and data import

```
# Load in data from Figure 4
## Human ERP
hum_erp4_aovdat <- readxl::read_xlsx("Figure4_data.xlsx", sheet = 1)
hum_erp4_aovdat$response <- factor(hum_erp4_aovdat$response, levels = c("Nontarget", "Target"))
hum_erp4_aovdat$reward <- factor(hum_erp4_aovdat$reward, levels = c("Nonreward", "Reward"))
hum_erp4_aovdat$trial_type <- factor(hum_erp4_aovdat$trial_type, levels = c("NTNR", "NTR", "TR", "TNR"))
hum_erp4_aovdat$treat <- factor(hum_erp4_aovdat$treat, levels = c("placebo", "low", "high"))

## Human Pe
hum_pe4_aovdat <- readxl::read_xlsx("Figure4_data.xlsx", sheet = 2)
hum_pe4_aovdat$response <- factor(hum_pe4_aovdat$response, levels = c("Nontarget", "Target"))
hum_pe4_aovdat$reward <- factor(hum_pe4_aovdat$reward, levels = c("Nonreward", "Reward"))
hum_pe4_aovdat$trial_type <- factor(hum_pe4_aovdat$trial_type, levels = c("NTNR", "NTR", "TR", "TNR"))
hum_pe4_aovdat$treat <- factor(hum_pe4_aovdat$treat, levels = c("placebo", "low", "high"))

  
## Rodent ERP
rat_erp4_aovdat <- readxl::read_xlsx("Figure4_data.xlsx", sheet = 3)
rat_erp4_aovdat$response <- factor(rat_erp4_aovdat$response)
rat_erp4_aovdat$reward <- factor(rat_erp4_aovdat$reward)
rat_erp4_aovdat$trial_type <- factor(rat_erp4_aovdat$trial_type, levels = c("NTNR", "NTR", "TR", "TNR"))
rat_erp4_aovdat$treat <- factor(rat_erp4_aovdat$treat, levels = c("DMSO", "2mg", "4mg", "8mg", "16mg", "32mg", "64mg"))


## Rodent PE
rat_pe4_aovdat <- readxl::read_xlsx("Figure4_data.xlsx", sheet = 4)
rat_pe4_aovdat$response <- factor(rat_pe4_aovdat$response, levels = c("Nontarget", "Target"))
rat_pe4_aovdat$reward <- factor(rat_pe4_aovdat$reward, levels = c("Nonreward", "Reward"))
rat_pe4_aovdat$trial_type <- factor(rat_pe4_aovdat$trial_type, levels = c("NTNR", "NTR", "TR", "TNR"))
rat_pe4_aovdat$treat <- factor(rat_pe4_aovdat$treat, levels = c("DMSO", "2mg", "4mg", "8mg", "16mg", "32mg", "64mg"))
```

### 1.2.2 - ERPs

```
# Human "2-way" Model (has to be 1-way)
hum_plac_erp <- hum_erp4_aovdat %>%
                  filter(treat == "placebo") %>%
                    filter(trial_type == "TR" | trial_type == "TNR")

hum_erp_m3 <- aov_ez("subject", "ERP_win", hum_plac_erp, within = c("reward")); hum_erp_m3


# Rodent "2-way" Model (has to be 1-way)
rat_plac_erp <- rat_erp4_aovdat %>%
                  filter(treat == "DMSO") %>%
                    filter(trial_type == "TR" | trial_type == "TNR")

rat_erp_m3 <- aov_ez("subject", "ERP_win", rat_plac_erp, within = c("reward")); rat_erp_m3
```

### 1.2.3 - PE

```
# Human "2-way" Model (has to be 1-way)
hum_plac_pe <- hum_pe4_aovdat %>%
                  filter(treat == "placebo") %>%
                    filter(trial_type == "TR" | trial_type == "TNR")

hum_pe_m3 <- aov_ez("subject", "PE", hum_plac_pe, within = c("reward")); hum_pe_m3


# Rodent "2-way" Model (has to be 1-way)
rat_plac_pe <- rat_pe4_aovdat %>%
                  filter(treat == "DMSO") %>%
                    filter(trial_type == "TR" | trial_type == "TNR")

rat_pe_m3 <- aov_ez("subject", "PE", rat_plac_pe, within = c("reward")); rat_pe_m3


# Rodent 1-way Model across all treats
rat_rew_pe <- rat_pe4_aovdat %>%
                filter(reward == "Reward") %>%
                  filter(trial_type == "TR" | trial_type == "TNR")

rat_pe_m4 <- aov_ez("subject", "PE", rat_rew_pe, within = "treat"); rat_pe_m4

rat_unrew_pe <- rat_pe4_aovdat %>%
                  filter(reward == "Nonreward") %>%
                    filter(trial_type == "TR" | trial_type == "TNR")

rat_pe_m5 <- aov_ez("subject", "PE", rat_unrew_pe, within = "treat"); rat_pe_m5
```

# 2.0 - Boxplot Figures

## 2.1 - Figure 1

```
fig1_hum_revs <- read_excel("./Figure1_data.xlsx", sheet = 1)
fig1_hum_perf <- read_excel("./Figure1_data.xlsx", sheet = 2)
fig1_hum_perf$var = factor(fig1_hum_perf$var, levels = c("TWS", "TLS"))

fig1_rod_revs <- read_excel("./Figure1_data.xlsx", sheet = 3)
fig1_rod_perf <- read_excel("./Figure1_data.xlsx", sheet = 4)
fig1_rod_perf$var = factor(fig1_rod_perf$var, levels = c("TWS", "TLS"))

# Human Revs Plot
hum_revs_plot <- ggplot(fig1_hum_revs, aes(x = 0, y = revs_per_100)) +
  geom_boxplot(fill = "grey", alpha = 0.8, outliers = TRUE,  linewidth = 0.5, width = 0.5) +
  stat_summary(fun.y = mean, geom="point", shape = 17, size = 2.5) +
  stat_summary(fun.data = mean_se, geom = "errorbar", width = 0.25) + 
  theme_classic(base_family = "sans") +
  theme(axis.title = element_text(size = 18), 
        axis.title.x = element_text(size = 18, color = "black"),
        axis.text.x = element_blank(),
        axis.ticks.x = element_blank(),
        axis.title.y = element_text(size = 18, colour = "black"),
        axis.text.y = element_text(size = 16, colour = "black"),
        legend.title=element_text(size = 12),
        legend.text = element_text(size = 10),
        legend.position = "none") +
        scale_y_continuous(breaks = c(0,2,4,6,8), limits = c(0,9)) +
        ylab("Reversals per 100 Trials") +
        xlab("Reversals"); hum_revs_plot

#pdf(file = "fig1_hum_revs_plot.pdf", width = 2.5, height = 5)
#hum_revs_plot
#dev.off()


# Human TWS/TLS Plot
hum_twls_plot <- ggplot(data=fig1_hum_perf, aes(x = var, y = probability)) +
  geom_boxplot(alpha = 0.8, outliers = TRUE, linewidth = 0.5, width = 0.5) +
  stat_summary(fun.y = mean, geom = "point", shape = 17, size = 2.5) +
  stat_summary(fun.data = mean_se, geom = "errorbar", width = 0.25) + 
  theme_classic(base_family = "sans") +
  theme(axis.title = element_text(size = 18), 
        axis.title.x = element_blank(),
        axis.text.x = element_text(size = 16, colour = "black"),
        axis.title.y = element_text(size = 18, colour = "black"),
        axis.text.y = element_text(size = 16, colour = "black"),
        legend.title=element_text(size = 12),
        legend.text = element_text(size = 10),
        legend.position = "none") +
        scale_y_continuous(breaks = c(0,0.2,0.4,0.6,0.8,1.0), position = "right") + 
        ylab("Probability"); hum_twls_plot

#pdf(file = "fig1_hum_twls_plot.pdf", width = 3.5, height = 5)
#hum_twls_plot 
#dev.off()


# Rodent Revs Plot
rod_revs_plot <- ggplot(fig1_rod_revs, aes(x = 0, y = revs_per_100)) +
  geom_boxplot(fill = "grey", alpha = 0.8, outliers = TRUE,  linewidth = 0.5, width = 0.5) +
  stat_summary(fun.y = mean, geom="point", shape = 17, size = 2.5) +
  stat_summary(fun.data = mean_se, geom = "errorbar", width = 0.25) + 
  theme_classic(base_family = "sans") +
  theme(axis.title = element_text(size = 18), 
        axis.title.x = element_text(size = 18, color = "black"),
        axis.text.x = element_blank(),
        axis.ticks.x = element_blank(),
        axis.title.y = element_text(size = 18, colour = "black"),
        axis.text.y = element_text(size = 16, colour = "black"),
        legend.title=element_text(size = 12),
        legend.text = element_text(size = 10),
        legend.position = "none") +
        scale_y_continuous(breaks = c(0,2,4,6,8), limits = c(0,9)) +
        ylab("Reversals per 100 Trials") +
        xlab("Reversals"); rod_revs_plot

#pdf(file = "fig_1rod_revs_plot.pdf", width = 2.5, height = 5) 
#rod_revs_plot 
#dev.off()


# Rodent Perf Plot
rod_twls_plot <- ggplot(data=fig1_rod_perf, aes(x = var, y = probability)) +
  geom_boxplot(alpha = 0.8, outliers = TRUE, linewidth = 0.5, width = 0.5) +
  stat_summary(fun.y = mean, geom = "point", shape = 17, size = 2.5) +
  stat_summary(fun.data = mean_se, geom = "errorbar", width = 0.25) + 
  theme_classic(base_family = "sans") +
  theme(axis.title = element_text(size = 18), 
        axis.title.x = element_blank(),
        axis.text.x = element_text(size = 16, colour = "black"),
        axis.title.y = element_text(size = 18, colour = "black"),
        axis.text.y = element_text(size = 16, colour = "black"),
        legend.title=element_text(size = 12),
        legend.text = element_text(size = 10),
        legend.position = "none") +
        scale_y_continuous(breaks = c(0,0.2,0.4,0.6,0.8,1.0), position = "right") + 
        ylab("Probability"); rod_twls_plot

#pdf(file = "fig1_rod_twls_plot.pdf", width = 3.5, height = 5)
#rod_twls_plot
#dev.off()
```

## 2.2 - Figure 2

```
fig2_hum_erp <- read_excel("./Figure2_data.xlsx", sheet = 1)
fig2_hum_erp$response_type <- factor(fig2_hum_erp$trial_type, levels = c("TNR", "NTNR", "TR", "NTR"), labels = c("Target No Reward", "NonTarget No Reward", "Target Reward", "NonTarget Reward"))

fig2_hum_PE <- read_excel("./Figure2_data.xlsx", sheet = 2)
fig2_hum_PE$response_type <- factor(fig2_hum_PE$trial_type, levels = c("TNR", "NTNR", "TR", "NTR"), labels = c("Target No Reward", "NonTarget No Reward", "Target Reward", "NonTarget Reward"))

fig2_rod_erp <- read_excel("./Figure2_data.xlsx", sheet = 3)
fig2_rod_erp$response_type <- factor(fig2_rod_erp$trial_type, levels = c("TNR", "NTNR", "TR", "NTR"), labels = c("Target No Reward", "NonTarget No Reward", "Target Reward", "NonTarget Reward"))

fig2_rod_PE <- read_excel("./Figure2_data.xlsx", sheet = 4)
fig2_rod_PE$response_type <- factor(fig2_rod_PE$trial_type, levels = c("TNR", "NTNR", "TR", "NTR"), labels = c("Target No Reward", "NonTarget No Reward", "Target Reward", "NonTarget Reward"))


# Human ERP
hum_erp_plot <- ggplot(fig2_hum_erp, aes(x = trial_type, y = ERP_win, fill = trial_type)) +
  geom_boxplot(alpha = 0.8, outliers = TRUE,  linewidth = 0.5, width = 0.5) +
  stat_summary(fun.y = mean, geom="point", shape = 17, size = 2.5) +
  stat_summary(fun.data = mean_se, geom = "errorbar", width = 0.25) + 
  theme_classic(base_family = "sans") +
  theme(axis.title = element_text(size = 18), 
        axis.title.x = element_text(size = 18, color = "black"),
        axis.text.x = element_text(size = 16, color = "black"),
        axis.title.y = element_text(size = 18, colour = "black"),
        axis.text.y = element_text(size = 16, colour = "black"),
        legend.title=element_text(size = 12),
        legend.text = element_text(size = 10),
        legend.position = "none") +
        scale_y_continuous(breaks = c(-6,-3,0,3,6,9,12), limits = c(-6,13)) +
        ylab("µV") +
        xlab("Response Type"); hum_erp_plot

#pdf(file = "fig2_hum_erp_plot.pdf", width = 5, height = 4)
#hum_erp_plot
#dev.off()

# Human PE
hum_pe_plot <- ggplot(fig2_hum_PE, aes(x = trial_type, y = PE, fill = trial_type)) +
  geom_boxplot(alpha = 0.8, outliers = TRUE,  linewidth = 0.5, width = 0.5) +
  stat_summary(fun.y = mean, geom="point", shape = 17, size = 2.5) +
  stat_summary(fun.data = mean_se, geom = "errorbar", width = 0.25) + 
  geom_hline(yintercept=0) +
  theme_classic(base_family = "sans") +
  theme(axis.title = element_text(size = 18), 
        axis.title.x = element_text(size = 18, color = "black"),
        axis.text.x = element_text(size = 16, color = "black"),
        axis.title.y = element_text(size = 18, colour = "black"),
        axis.text.y = element_text(size = 16, colour = "black"),
        legend.title=element_text(size = 12),
        legend.text = element_text(size = 10),
        legend.position = "none") +
        scale_y_continuous(breaks = c(-1,-0.5,0,0.5,1), limits = c(-1,1)) +
        ylab("PE Value") +
        xlab("Response Type"); hum_pe_plot

#pdf(file = "fig2_hum_pe_plot.pdf", width = 5, height = 4)
#hum_pe_plot
#dev.off()

# Rodent ERP
rod_erp_plot <- ggplot(fig2_rod_erp, aes(x = trial_type, y = ERP_win, fill = trial_type)) +
  geom_boxplot(alpha = 0.8, outliers = TRUE,  linewidth = 0.5, width = 0.5) +
  stat_summary(fun.y = mean, geom="point", shape = 17, size = 2.5) +
  stat_summary(fun.data = mean_se, geom = "errorbar", width = 0.25) + 
  theme_classic(base_family = "sans") +
  theme(axis.title = element_text(size = 18), 
        axis.title.x = element_text(size = 18, color = "black"),
        axis.text.x = element_text(size = 16, color = "black"),
        axis.title.y = element_text(size = 18, colour = "black"),
        axis.text.y = element_text(size = 16, colour = "black"),
        legend.title=element_text(size = 12),
        legend.text = element_text(size = 10),
        legend.position = "none") +
        scale_y_continuous(breaks = c(-40,-30,-20,-10,0,10,20,30,40,50), limits = c(-40,52)) +
        ylab("µV") +
        xlab("Response Type"); rod_erp_plot

#pdf(file = "fig2_rod_erp_plot.pdf", width = 5, height = 4)
#rod_erp_plot
#dev.off()

# Rodent PE
rod_pe_plot <- ggplot(fig2_rod_PE, aes(x = trial_type, y = PE, fill = trial_type)) +
  geom_boxplot(alpha = 0.8, outliers = TRUE,  linewidth = 0.5, width = 0.5) +
  stat_summary(fun.y = mean, geom="point", shape = 17, size = 2.5) +
  stat_summary(fun.data = mean_se, geom = "errorbar", width = 0.25) + 
  geom_hline(yintercept=0) +
  theme_classic(base_family = "sans") +
  theme(axis.title = element_text(size = 18), 
        axis.title.x = element_text(size = 18, color = "black"),
        axis.text.x = element_text(size = 16, color = "black"),
        axis.title.y = element_text(size = 18, colour = "black"),
        axis.text.y = element_text(size = 16, colour = "black"),
        legend.title=element_text(size = 12),
        legend.text = element_text(size = 10),
        legend.position = "none") +
        scale_y_continuous(breaks = c(-1,-0.5,0,0.5,1), limits = c(-1,1)) +
        ylab("µV") +
        xlab("Response Type"); rod_pe_plot

#pdf(file = "fig2_rod_pe_plot.pdf", width = 5, height = 4)
#rod_pe_plot
#dev.off()
```

## 2.3 - Figure 4

```
fig4_hum_erp <- read_excel("Figure4_plotdata.xlsx", sheet = 1)
fig4_hum_erp$response_type <- factor(fig4_hum_erp$response_type, levels = c("TR", "TNR"), labels = c("Target Reward", "Target No Reward"))
fig4_hum_erp$dose <- factor(fig4_hum_erp$dose, levels = c("placebo","low","high"), labels = c("0","100", "200"))

fig4_hum_pe <- read_excel("Figure4_plotdata.xlsx", sheet = 2)
fig4_hum_pe$response_type <- factor(fig4_hum_pe$response_type, levels = c("TR", "TNR"), labels = c("Target Reward", "Target No Reward"))
fig4_hum_pe$dose <- factor(fig4_hum_pe$dose, levels = c("placebo","low","high"), labels = c("0","100", "200"))

fig4_rod_erp <- read_excel("Figure4_plotdata.xlsx", sheet = 3)
fig4_rod_erp$response_type <- factor(fig4_rod_erp$response_type, levels = c("TR", "TNR"), labels = c("Target Reward", "Target No Reward"))
fig4_rod_erp$dose <- factor(fig4_rod_erp$dose, levels = c("DMSO","4mg","8mg", "16mg", "32mg", "64mg"), labels = c("0","4","8","16","32","64"))

fig4_rod_pe <- read_excel("Figure4_plotdata.xlsx", sheet = 4)
fig4_rod_pe$response_type <- factor(fig4_rod_pe$response_type, levels = c("TR", "TNR"), labels = c("Target Reward", "Target No Reward"))
fig4_rod_pe$dose <- factor(fig4_rod_pe$dose, levels = c("DMSO","4mg","8mg", "16mg", "32mg", "64mg"), labels = c("0","4","8","16","32","64"))


# Human ERP
hum_erp_plot2 <- ggplot(fig4_hum_erp, aes(x = dose, y = ERP_win, fill = response_type)) +
  geom_boxplot(alpha = 0.8, outliers = TRUE,  linewidth = 0.5, width = 0.5) +
  stat_summary(fun.y = mean, geom="point", shape = 17, size = 2.5) +
  stat_summary(fun.data = mean_se, geom = "errorbar", width = 0.25) + 
  theme_classic(base_family = "sans") +
  theme(axis.title = element_text(size = 18), 
        axis.title.x = element_text(size = 18, color = "black"),
        axis.text.x = element_text(size = 16, color = "black"),
        axis.title.y = element_text(size = 18, colour = "black"),
        axis.text.y = element_text(size = 16, colour = "black"),
        legend.title=element_text(size = 12),
        legend.text = element_text(size = 10),
        legend.position = "none") +
        scale_y_continuous(breaks = c(-6,-3,0,3,6,9,12), limits = c(-6,12)) +
        ylab("µV") +
        xlab("Modafinil Dose (mg)") +
  facet_wrap(~response_type); hum_erp_plot2

#pdf(file = "fig4_hum_erp_plot.pdf", width = 5, height = 4)
#hum_erp_plot2
#dev.off()

# Human PE plot
hum_pe_plot2 <- ggplot(fig4_hum_pe, aes(x = dose, y = PE, fill = response_type)) +
  geom_boxplot(alpha = 0.8, outliers = TRUE,  linewidth = 0.5, width = 0.5) +
  stat_summary(fun.y = mean, geom="point", shape = 17, size = 2.5) +
  stat_summary(fun.data = mean_se, geom = "errorbar", width = 0.25) + 
  geom_hline(yintercept=0) +
  theme_classic(base_family = "sans") +
  theme(axis.title = element_text(size = 18), 
        axis.title.x = element_text(size = 18, color = "black"),
        axis.text.x = element_text(size = 16, color = "black"),
        axis.title.y = element_text(size = 18, colour = "black"),
        axis.text.y = element_text(size = 16, colour = "black"),
        legend.title=element_text(size = 12),
        legend.text = element_text(size = 10),
        legend.position = "none") +
        scale_y_continuous(breaks = c(-1,-0.5,0,0.5,1), limits = c(-1,1)) +
        ylab("PE Value") +
        xlab("Modafinil Dose (mg)") +
  facet_wrap(~response_type); hum_pe_plot2

pdf(file = "fig4_hum_pe_plot.pdf", width = 5, height = 4)
hum_pe_plot2
dev.off()

# Rod ERP Plot
rod_erp_plot2 <- ggplot(fig4_rod_erp, aes(x = dose, y = ERP_win, fill = response_type)) +
  geom_boxplot(alpha = 0.8, outliers = TRUE,  linewidth = 0.5, width = 0.5) +
  stat_summary(fun.y = mean, geom="point", shape = 17, size = 2.5) +
  stat_summary(fun.data = mean_se, geom = "errorbar", width = 0.25) + 
  theme_classic(base_family = "sans") +
  theme(axis.title = element_text(size = 18), 
        axis.title.x = element_text(size = 18, color = "black"),
        axis.text.x = element_text(size = 16, color = "black"),
        axis.title.y = element_text(size = 18, colour = "black"),
        axis.text.y = element_text(size = 16, colour = "black"),
        legend.title=element_text(size = 12),
        legend.text = element_text(size = 10),
        legend.position = "none") +
        scale_y_continuous(breaks = c(-50,-25,0,25,50), limits = c(-50,50)) +
        ylab("µV") +
        xlab("Modafinil Dose (mg)") +
  facet_wrap(~response_type); rod_erp_plot2

#pdf(file = "fig4_rod_erp_plot.pdf", width = 5, height = 4)
#rod_erp_plot2
#dev.off()

# Rod PE Plot
rod_pe_plot2 <- ggplot(fig4_rod_pe, aes(x = dose, y = PE, fill = response_type)) +
  geom_boxplot(alpha = 0.8, outliers = TRUE,  linewidth = 0.5, width = 0.5) +
  stat_summary(fun.y = mean, geom="point", shape = 17, size = 2.5) +
  stat_summary(fun.data = mean_se, geom = "errorbar", width = 0.25) + 
  geom_hline(yintercept=0) +
  theme_classic(base_family = "sans") +
  theme(axis.title = element_text(size = 18), 
        axis.title.x = element_text(size = 18, color = "black"),
        axis.text.x = element_text(size = 16, color = "black"),
        axis.title.y = element_text(size = 18, colour = "black"),
        axis.text.y = element_text(size = 16, colour = "black"),
        legend.title=element_text(size = 12),
        legend.text = element_text(size = 10),
        legend.position = "none") +
        scale_y_continuous(breaks = c(-1,-0.5,0,0.5,1), limits = c(-1,1)) +
        ylab("PE Value") +
        xlab("Modafinil Dose (mg)") +
  facet_wrap(~response_type); rod_pe_plot2

#pdf(file = "fig4_rod_pe_plot.pdf", width = 5, height = 4)
#rod_pe_plot2
#dev.off()
```
