# Supplementary material for: Identification of conserved frontal neurophysiological markers of cognitive flexibility in humans and rats
Source: Commun Biol. 2025 Aug 23;8:1268. doi: 10.1038/s42003-025-08729-x (PMC12375130; doi:10.1038/s42003-025-08729-x)
Supplement: Supplementary file 6 — Python code for RL model fitting and testing [file 42003_2025_8729_MOESM6_ESM.html]

Final\_RL\_models


In [ ]:

```
import os
import numpy as np
import pandas as pd
import seaborn as sns
import scipy as sp
import matplotlib.pyplot as plt
import getpass
import math
import re
import random
import warnings
warnings.filterwarnings('ignore')
```

# Load raw behavior¶

In [ ]:

```
base_dir = 'C:/Users/samba/Dropbox/Work Folder/Python Scripts/PRL/UH3_Project/Submission 2/Rat/'

path2data = base_dir + 'Data/'
save_path = base_dir + 'Analysis/'
```

In [ ]:

```
df = pd.read_csv(path2data + 'Rodent_PRL_UH23_study.csv')
df = df.dropna(axis=1, how='all')
df.reset_index(inplace=True, drop=True)
```

## Preprocessing data¶

In [ ]:

```
def CodeLeverPress(data):       
    '''
    get lever pressed and side selected. TargetSide (1, 4) indicates whether the left or right lever was the target. The value of the tar or nontar variable
    identifies the outcome (1=reward, -1=noreward, 0=other response selected)
    ''' 
    tar, nontar, TarSide = data
    if tar != 0 and TarSide == 1:
        resp = 'target'
        side = 'L' 
        action = 0
    elif tar != 0 and TarSide == 4:
        resp = 'target'
        side = 'R' 
        action = 1
    elif nontar != 0 and TarSide == 4:
        resp = 'non_target'
        side = 'L'  
        action = 0
    elif nontar != 0 and TarSide == 1:
        resp = 'non_target'
        side = 'R' 
        action = 1
    else:
        resp = 'none'
        side = 'none'
        action = 'none'
    return (resp, side, action)
```

In [ ]:

```
def CodeFdbk(data):
    '''Code feedback as reward or zero.'''
    to, nto = data
    if to == -1 or nto == -1:
        outcome = 'zero'
    elif to == 1 or nto == 1:
        outcome = 'reward'
    else:
        outcome = 'none'

    return outcome
```

In [ ]:

```
df['response'],df['SidePressed'],df['action'] = zip(*df[['TargetOutcome', 'NonTargetOutcome', 'TargetSide']].apply(CodeLeverPress,axis=1))
df['outcome'] = df[['TargetOutcome','NonTargetOutcome']].apply(CodeFdbk,axis=1)
df['reward'] = df[['TargetOutcome','NonTargetOutcome']].apply(CodeFdbk,axis=1)

df.reward.replace({'reward':1,'zero':0},inplace=True)
```

In [ ]:

```
mod_data = df
```

# Model Functions¶

In [ ]:

```
def softmax(QA, QB, beta):
    Q = np.array((QA, QB))
    num = np.exp(Q * beta)
    den = np.exp(Q * beta).sum()
        
    return num / den

def softmaxBias(QA, QB, beta, bias):
    Q = np.array((QA + bias, QB))
    num = np.exp(Q * beta)
    den = np.exp(Q * beta).sum()
        
    return num / den

def GetBIC(ll,nparams,ntrials):  
    BIC = -2.0 * ll + np.log(ntrials) * nparams 
    return BIC
```

In [ ]:

```
def Start_Params_with_Bounds(fit_param_list, bounds_list):   
 
    start_params = []
    
    for i in range(len(fit_param_list)):
        if  bounds_list[i][0] == 0 and bounds_list[i][1] == 1:    
            num = random.uniform(0.001, 0.999)
        elif bounds_list[i][0] == 0 and bounds_list[i][1] == 10:  
            num = random.uniform(0.001, 9.999)
        elif bounds_list[i][0] == -1 and bounds_list[i][1] == 1:  
            num = random.uniform(-0.999, 0.999)
        else:
            print('incomplete bound range')
        start_params.append(num)
        
    return start_params
```

In [ ]:

```
def Fit_Function(df, model, QLearn_like, bounds_list, fit_param_list, num_iters):
    
    print(model) # to confirm desired model is ran
    
    neg_ll = 999999999   
    optimized = False   
    
    for random_start in range(num_iters):
    
        start_params = Start_Params_with_Bounds(fit_param_list, bounds_list)
              
        opt_new = sp.optimize.minimize(QLearn_like, 
                                       [start_params],
                                       args=(df, model),
                                       bounds = bounds_list,
                                       method='TNC', 
                                       tol=1e-5,
                                       options={'disp':True, 'maxiter':5000, 'eps':1e-8, 'ftol':1e-8, 'gtol':1e-8})
        prog = [opt_new.fun]
        prog.extend(opt_new.x[0:])

        if opt_new.fun < neg_ll:  
            neg_ll = opt_new.fun
            ll = -1*neg_ll
            for i in range(len(fit_param_list)):  
                fit_param_list[i] = opt_new.x[i]
            optimized = opt_new.success
            
        if optimized == False:
            print('subject = ' + str(df.subject.unique()[0]) + ', optimization failed!')
        
    result_list = [ll]
    result_list.extend(fit_param_list)

    return result_list

num_iters = 20
```

In [ ]:

```
def OneAlpha_like(params,df, model):

    alpha, beta = params
    
    Q = {0:0.5,1:0.5}
        
    prob_log = 0
    i = 0

    for i, row_i in df.iterrows():
        chosen = row_i.action
        for action in Q.keys():
            if action != chosen:
                not_chosen = action
                
        Qc = Q[chosen]
        Qnc = Q[not_chosen]
        QA = Q[0]
        QB = Q[1]
        
        probs = softmax(QA, QB, beta)[chosen]
        prob_log += np.log(softmax(QA, QB, beta)[chosen])    
        pA = softmax(QA, QB, beta)[0]
        pB = softmax(QA, QB, beta)[1]
                          
        fdbk = row_i.reward
        PE = fdbk - Qc
        Q_new = Qc + alpha*PE
            
        Q[chosen] = Q_new
        Q[not_chosen] = Qnc
                
    return -prob_log


def OneAlpha_return_df(a, b, df):

    alpha = a
    beta = b
    
    Q = {0:0.5,1:0.5}
        
    prob_log = 0
    i = 0
    
    for i, row_i in df.iterrows():
        chosen = row_i.action
        for action in Q.keys():
            if action != chosen:
                not_chosen = action
                
        Qc = Q[chosen]
        Qnc = Q[not_chosen]
        QA = Q[0]
        QB = Q[1]
        
        probs = softmax(QA, QB, beta)[chosen]
        prob_log += np.log(softmax(QA, QB, beta)[chosen])    
        pA = softmax(QA, QB, beta)[0]
        pB = softmax(QA, QB, beta)[1]
  
        fdbk = row_i.reward   
        PE = fdbk - Qc
        Q_new = Qc + alpha*PE

        Q[chosen] = Q_new
        Q[not_chosen] = Qnc
        
        df.loc[i, 'Q_Chosen'] = Qc
        df.loc[i, 'Q_NotChosen'] = Qnc
        df.loc[i, 'Q_Chosen_updated'] = Q_new
        df.loc[i, 'Q_left'] = QA
        df.loc[i, 'Q_right'] = QB
        df.loc[i, 'pA'] = pA
        df.loc[i, 'pB'] = pB
        df.loc[i, 'PE'] = PE             

    return df
```

In [ ]:

```
def OneAlphaDoubleUpdate_like(params,df, model):

    alpha, beta = params

    Q = {0:0.5,1:0.5}
        
    prob_log = 0
    i = 0

    for i, row_i in df.iterrows():
        chosen = row_i.action
        for action in Q.keys():
            if action != chosen:
                not_chosen = action
                
        Qc = Q[chosen]
        Qnc = Q[not_chosen]
        QA = Q[0]
        QB = Q[1]
        
        probs = softmax(QA, QB, beta)[chosen]
        prob_log += np.log(softmax(QA, QB, beta)[chosen])    
        pA = softmax(QA, QB, beta)[0]
        pB = softmax(QA, QB, beta)[1]
                           
        fdbk = row_i.reward
        PE = fdbk - Qc
        Q_new = Qc + alpha*PE
        Q_nc_new = Qnc - alpha*PE
            
        Q[chosen] = Q_new
        Q[not_chosen] = Q_nc_new
                
    return -prob_log


def OneAlphaDoubleUpdate_return_df(a, b, df):

    alpha = a
    beta = b
    
    Q = {0:0.5,1:0.5}
        
    prob_log = 0
    i = 0

    for i, row_i in df.iterrows():
        chosen = row_i.action
        for action in Q.keys():
            if action != chosen:
                not_chosen = action
                
        Qc = Q[chosen]
        Qnc = Q[not_chosen]
        QA = Q[0]
        QB = Q[1]
        
        probs = softmax(QA, QB, beta)[chosen]
        prob_log += np.log(softmax(QA, QB, beta)[chosen])    
        pA = softmax(QA, QB, beta)[0]
        pB = softmax(QA, QB, beta)[1]
                          
        fdbk = row_i.reward  
        PE = fdbk - Qc
        Q_new = Qc + alpha*PE
        Q_nc_new = Qnc - alpha*PE
            
        Q[chosen] = Q_new
        Q[not_chosen] = Q_nc_new
        
        df.loc[i, 'Q_Chosen'] = Qc
        df.loc[i, 'Q_NotChosen'] = Qnc
        df.loc[i, 'Q_Chosen_updated'] = Q_new
        df.loc[i, 'Q_NotChosen_updated'] = Q_nc_new
        df.loc[i, 'Q_left'] = QA
        df.loc[i, 'Q_right'] = QB
        df.loc[i, 'pA'] = pA
        df.loc[i, 'pB'] = pB
        df.loc[i, 'PE'] = PE             

    return df
```

In [ ]:

```
def OneAlphaForget_like(params,df, model):
 
    alpha, beta, forget = params
    
    Q = {0: 0.5, 1: 0.5}
    
    prob_log = 0
    i = 0

    for i, row_i in df.iterrows():
        chosen = row_i.action
        for action in Q.keys():
            if action != chosen:
                not_chosen = action
                
        Qc, Qnc = Q[chosen], Q[not_chosen]
        QA, QB = Q[0], Q[1]

        probs = softmax(QA, QB, beta)
        prob_log += np.log(probs[chosen]) 
        pA, pB = probs[0], probs[1]
            
        fdbk = row_i.reward
        PE = fdbk - Qc

        Q_new = Qc + alpha*PE
        Q_nc_new = (1-forget) * Qnc

        Q[chosen] = Q_new
        Q[not_chosen] = Q_nc_new

    prob_log *= -1
    return prob_log


def OneAlphaForget_return_df(best_a, best_b, best_f, df):
        
    alpha = best_a
    beta = best_b
    forget = best_f

    Q = {0: 0.5, 1: 0.5}
    
    prob_log = 0
    i = 0

    for i, row_i in df.iterrows():
        chosen = row_i.action
        for action in Q.keys():
            if action != chosen:
                not_chosen = action
                
        Qc, Qnc = Q[chosen], Q[not_chosen]
        QA, QB = Q[0], Q[1]
        
        probs = softmax(QA, QB, beta)
        prob_log += np.log(probs[chosen]) 
        pA, pB = probs[0], probs[1]
            
        fdbk = row_i.reward
        PE = fdbk - Qc
        
        Q_new = Qc + alpha*PE
        Q_nc_new = (1-forget) * Qnc
            
        Q[chosen] = Q_new
        Q[not_chosen] = Q_nc_new

        df.loc[i, 'Q_Chosen'] = Qc
        df.loc[i, 'Q_NotChosen'] = Qnc
        df.loc[i, 'Q_Chosen_updated'] = Q_new
        df.loc[i, 'Q_NotChosen_updated'] = Q_nc_new
        df.loc[i, 'Q_left'] = QA
        df.loc[i, 'Q_right'] = QB
        df.loc[i, 'pA'] = pA
        df.loc[i, 'pB'] = pB
        df.loc[i, 'PE'] = PE             
        
    return df
```

In [ ]:

```
def OneAlphaBias_like(params,df, model):

    alpha, beta, bias = params
    
    Q = {0:0.5,1:0.5}
    
    prob_log = 0
    i = 0

    for i, row_i in df.iterrows():
        chosen = row_i.action
        for action in Q.keys():
            if action != chosen:
                not_chosen = action
                
        Qc = Q[chosen]
        Qnc = Q[not_chosen]
        QA = Q[0]
        QB = Q[1]
    
        probs = softmaxBias(QA, QB, beta, bias)[chosen]
        prob_log += np.log(softmaxBias(QA, QB, beta, bias)[chosen])    
        pA = softmaxBias(QA, QB, beta, bias)[0]
        pB = softmaxBias(QA, QB, beta, bias)[1]
        
        fdbk = row_i.reward
        PE = fdbk - Qc
        Q_new = Qc + alpha*PE

        Q[chosen] = Q_new
        Q[not_chosen] = Qnc

    return -prob_log

def OneAlphaBias_return_df(best_a, best_b, best_bias, df):
        
    alpha = best_a
    beta = best_b
    bias = best_bias

    Q = {0:0.5,1:0.5}
    
    prob_log = 0
    i = 0

    for i, row_i in df.iterrows():
        chosen = row_i.action
        for action in Q.keys():
            if action != chosen:
                not_chosen = action
                
        Qc = Q[chosen]
        Qnc = Q[not_chosen]
        QA = Q[0]
        QB = Q[1]
        
        probs = softmaxBias(QA, QB, beta, bias)[chosen]
        prob_log += np.log(softmaxBias(QA, QB, beta, bias)[chosen])    
        pA = softmax(QA, QB, beta)[0]
        pB = softmax(QA, QB, beta)[1]
        
        # Compute PE and update the chosen Q-value
        fdbk = row_i.reward
        PE = fdbk - Qc
        Q_new = Qc + alpha*PE
        Q[chosen] = Q_new

                 
        df.loc[i, 'Q_Chosen'] = Qc
        df.loc[i, 'Q_NotChosen'] = Qnc
        df.loc[i, 'Q_Chosen_updated'] = Q_new
        df.loc[i, 'Q_left'] = QA
        df.loc[i, 'Q_right'] = QB
        df.loc[i, 'pA'] = pA
        df.loc[i, 'pB'] = pB
        df.loc[i, 'PE'] = PE             
        
    return df
```

In [ ]:

```
def OneAlphaBiasForget_like(params,df, model):

    alpha, beta, bias, forget = params
    
    Q = {0:0.5,1:0.5}
    
    prob_log = 0
    i = 0

    for i, row_i in df.iterrows():
        chosen = row_i.action
        for action in Q.keys():
            if action != chosen:
                not_chosen = action
                
        Qc = Q[chosen]
        Qnc = Q[not_chosen]
        QA = Q[0]
        QB = Q[1]
    
        probs = softmaxBias(QA, QB, beta, bias)[chosen]
        prob_log += np.log(softmaxBias(QA, QB, beta, bias)[chosen])    
        pA = softmaxBias(QA, QB, beta, bias)[0]
        pB = softmaxBias(QA, QB, beta, bias)[1]

        fdbk = row_i.reward
        PE = fdbk - Qc
        Q_new = Qc + alpha*PE
        Q_nc_new = (1-forget) * Qnc

        Q[chosen] = Q_new
        Q[not_chosen] = Q_nc_new

    return -prob_log


def OneAlphaBiasForget_return_df(best_a, best_b, best_bias, best_forget, df):
        
    alpha = best_a
    beta = best_b
    bias = best_bias
    forget = best_forget

    Q = {0:0.5,1:0.5}
    
    prob_log = 0
    i = 0

    for i, row_i in df.iterrows():
        chosen = row_i.action
        for action in Q.keys():
            if action != chosen:
                not_chosen = action
                
        Qc = Q[chosen]
        Qnc = Q[not_chosen]
        QA = Q[0]
        QB = Q[1]
        
        probs = softmaxBias(QA, QB, beta, bias)[chosen]
        prob_log += np.log(softmaxBias(QA, QB, beta, bias)[chosen])    
        pA = softmax(QA, QB, beta)[0]
        pB = softmax(QA, QB, beta)[1]
        
        fdbk = row_i.reward
        PE = fdbk - Qc
        Q_new = Qc + alpha*PE
        Q_nc_new = (1-forget) * Qnc
        
        Q[chosen] = Q_new
        Q[not_chosen] = Q_nc_new
                 
        df.loc[i, 'Q_Chosen'] = Qc
        df.loc[i, 'Q_NotChosen'] = Qnc
        df.loc[i, 'Q_Chosen_updated'] = Q_new
        df.loc[i, 'Q_NotChosen_updated'] = Q_nc_new
        df.loc[i, 'Q_left'] = QA
        df.loc[i, 'Q_right'] = QB
        df.loc[i, 'pA'] = pA
        df.loc[i, 'pB'] = pB
        df.loc[i, 'PE'] = PE             
        
    return df
```

In [ ]:

```
def TwoAlpha_like(params,df,model):
    '''Update Q-values using the delta rule plus softmax. 
    one alpha learning rate.
    '''  
    alpha_pos, alpha_neg, beta = params

    Q = {0:0.5,1:0.5}
        
    prob_log = 0
    i = 0

    for i, row_i in df.iterrows():
        chosen = row_i.action
        for action in Q.keys():
            if action != chosen:
                not_chosen = action
                
        Qc = Q[chosen]
        Qnc = Q[not_chosen]
        QA = Q[0]
        QB = Q[1]
        
        probs = softmax(QA, QB, beta)[chosen]
        prob_log += np.log(softmax(QA, QB, beta)[chosen])    
        pA = softmax(QA, QB, beta)[0]
        pB = softmax(QA, QB, beta)[1]
   
        fdbk = row_i.reward
        
        PE = fdbk - Qc
        
        if fdbk == 0:
            Q_new = Qc + alpha_neg*PE   
        elif fdbk == 1:
            Q_new = Qc + alpha_pos*PE
            
        Q[chosen] = Q_new
        Q[not_chosen] = Qnc
                
    return -prob_log


def TwoAlpha_return_df(ag, al, b, df):
    '''Update Q-values using the delta rule plus softmax. 
    one alpha learning rate.
    '''  
    alpha_pos = ag 
    alpha_neg = al
    beta = b
    
    Q = {0:0.5,1:0.5}
        
    prob_log = 0
    i = 0

    for i, row_i in df.iterrows():
        chosen = row_i.action
        for action in Q.keys():
            if action != chosen:
                not_chosen = action
                
        Qc = Q[chosen]
        Qnc = Q[not_chosen]
        QA = Q[0]
        QB = Q[1]
        
        probs = softmax(QA, QB, beta)[chosen]
        prob_log += np.log(softmax(QA, QB, beta)[chosen])    
        pA = softmax(QA, QB, beta)[0]
        pB = softmax(QA, QB, beta)[1]
                          
        fdbk = row_i.reward      
        PE = fdbk - Qc
        
        if fdbk == 0:
            Q_new = Qc + alpha_neg*PE
            
        elif fdbk == 1:
            Q_new = Qc + alpha_pos*PE

        Q[chosen] = Q_new
        Q[not_chosen] = Qnc
        
        df.loc[i, 'Q_Chosen'] = Qc
        df.loc[i, 'Q_NotChosen'] = Qnc
        df.loc[i, 'Q_Chosen_updated'] = Q_new
        df.loc[i, 'Q_left'] = QA
        df.loc[i, 'Q_right'] = QB
        df.loc[i, 'pA'] = pA
        df.loc[i, 'pB'] = pB
        df.loc[i, 'PE'] = PE             

    return df
```

In [ ]:

```
def TwoAlphaDoubleUpdate_like(params,df,model):

    alpha_pos, alpha_neg, beta = params
    
    Q = {0:0.5,1:0.5}
        
    prob_log = 0
    i = 0

    for i, row_i in df.iterrows():
        chosen = row_i.action
        for action in Q.keys():
            if action != chosen:
                not_chosen = action
                
        Qc = Q[chosen]
        Qnc = Q[not_chosen]
        QA = Q[0]
        QB = Q[1]
        
        probs = softmax(QA, QB, beta)[chosen]
        prob_log += np.log(softmax(QA, QB, beta)[chosen])    
        pA = softmax(QA, QB, beta)[0]
        pB = softmax(QA, QB, beta)[1]
          
        fdbk = row_i.reward        
        PE = fdbk - Qc
        
        if fdbk == 0:
            Q_new = Qc + alpha_neg*PE
            Q_nc_new = Qnc - alpha_neg*PE
            
        elif fdbk == 1:
            Q_new = Qc + alpha_pos*PE
            Q_nc_new = Qnc - alpha_pos*PE
            
        Q[chosen] = Q_new
        Q[not_chosen] = Q_nc_new

    return -prob_log


def TwoAlphaDoubleUpdate_return_df(ag, al, b, df):

    alpha_pos = ag 
    alpha_neg = al
    beta = b
    
    Q = {0:0.5,1:0.5}
        
    prob_log = 0
    i = 0

    for i, row_i in df.iterrows():
        chosen = row_i.action
        for action in Q.keys():
            if action != chosen:
                not_chosen = action
                
        Qc = Q[chosen]
        Qnc = Q[not_chosen]
        QA = Q[0]
        QB = Q[1]
        
        probs = softmax(QA, QB, beta)[chosen]
        prob_log += np.log(softmax(QA, QB, beta)[chosen])    
        pA = softmax(QA, QB, beta)[0]
        pB = softmax(QA, QB, beta)[1]
                         
        fdbk = row_i.reward   
        PE = fdbk - Qc
        if fdbk == 0:
            Q_new = Qc + alpha_neg*PE
            Q_nc_new = Qnc - alpha_neg*PE
            
        elif fdbk == 1:
            Q_new = Qc + alpha_pos*PE
            Q_nc_new = Qnc - alpha_pos*PE

        Q[chosen] = Q_new
        Q[not_chosen] = Q_nc_new
               
        df.loc[i, 'Q_Chosen'] = Qc
        df.loc[i, 'Q_NotChosen'] = Qnc
        df.loc[i, 'Q_Chosen_updated'] = Q_new
        df.loc[i, 'Q_NotChosen_updated'] = Q_nc_new
        df.loc[i, 'Q_left'] = QA
        df.loc[i, 'Q_right'] = QB
        df.loc[i, 'pA'] = pA
        df.loc[i, 'pB'] = pB
        df.loc[i, 'PE'] = PE             

    return df
```

In [ ]:

```
def TwoAlphaForget_like(params,df, model):

    alpha_pos, alpha_neg, beta, forget = params
    
    Q = {0:0.5,1:0.5}
    
    prob_log = 0
    i = 0

    for i, row_i in df.iterrows():
        chosen = row_i.action
        for action in Q.keys():
            if action != chosen:
                not_chosen = action
                
        Qc = Q[chosen]
        Qnc = Q[not_chosen]
        QA = Q[0]
        QB = Q[1]
        
        probs = softmax(QA, QB, beta)[chosen]
        prob_log += np.log(softmax(QA, QB, beta)[chosen])    
        pA = softmax(QA, QB, beta)[0]
        pB = softmax(QA, QB, beta)[1]
                
        fdbk = row_i.reward
        PE = fdbk - Qc
        
        if fdbk == 0:
            Q_new = Qc + alpha_neg*PE
        elif fdbk == 1:
            Q_new = Qc + alpha_pos*PE
        
        Q_nc_new = (1-forget) * Qnc
                
        Q[chosen] = Q_new
        Q[not_chosen] = Q_nc_new

    prob_log *= -1
    return prob_log


def TwoAlphaForget_return_df(best_ag, best_al, best_b, best_forget, df):
        
    alpha_pos = best_ag
    alpha_neg = best_al
    beta = best_b
    forget = best_forget
    
    Q = {0:0.5,1:0.5}
    
    prob_log = 0
    i = 0

    for i, row_i in df.iterrows():
        chosen = row_i.action
        for action in Q.keys():
            if action != chosen:
                not_chosen = action
                
        Qc = Q[chosen]
        Qnc = Q[not_chosen]
        QA = Q[0]
        QB = Q[1]
        
        probs = softmax(QA, QB, beta)[chosen]
        prob_log += np.log(softmax(QA, QB, beta)[chosen])    
        pA = softmax(QA, QB, beta)[0]
        pB = softmax(QA, QB, beta)[1]
           
        fdbk = row_i.reward
        PE = fdbk - Qc
        
        if fdbk == 0:
            Q_new = Qc + alpha_neg*PE
            
        elif fdbk == 1:
            Q_new = Qc + alpha_pos*PE
            
        Q_nc_new = (1-forget) * Qnc

        Q[chosen] = Q_new
        Q[not_chosen] = Q_nc_new
                 
        df.loc[i, 'Q_Chosen'] = Qc
        df.loc[i, 'Q_NotChosen'] = Qnc
        df.loc[i, 'Q_Chosen_updated'] = Q_new
        df.loc[i, 'Q_NotChosen_updated'] = Q_nc_new
        df.loc[i, 'Q_left'] = QA
        df.loc[i, 'Q_right'] = QB
        df.loc[i, 'pA'] = pA
        df.loc[i, 'pB'] = pB
        df.loc[i, 'PE'] = PE             
        
    return df
```

In [ ]:

```
def TwoAlphaBias_like(params,df, model):

    alpha_pos, alpha_neg, beta, bias = params
    
    Q = {0:0.5,1:0.5}
    
    prob_log = 0
    i = 0

    for i, row_i in df.iterrows():
        chosen = row_i.action
        for action in Q.keys():
            if action != chosen:
                not_chosen = action

        Qc = Q[chosen]
        Qnc = Q[not_chosen]
        
        QA = Q[0]
        QB = Q[1]
        
        probs = softmaxBias(QA, QB, beta, bias)[chosen]
        prob_log += np.log(softmaxBias(QA, QB, beta, bias)[chosen])    
        pA = softmaxBias(QA, QB, beta, bias)[0]
        pB = softmaxBias(QA, QB, beta, bias)[1]
        
        fdbk = row_i.reward
        PE = fdbk - Qc
        
        if fdbk == 0:
            Q_new = Qc + alpha_neg*PE           
        elif fdbk == 1:
            Q_new = Qc + alpha_pos*PE

        Q[chosen] = Q_new

    prob_log *= -1
    return prob_log


def TwoAlphaBias_return_df(best_ag, best_al, best_b, best_bias, df):
        
    alpha_pos = best_ag
    alpha_neg = best_al
    beta = best_b
    bias = best_bias
    
    Q = {0:0.5,1:0.5}
    
    prob_log = 0
    i = 0
    
    for i, row_i in df.iterrows():
        chosen = row_i.action
        for action in Q.keys():
            if action != chosen:
                not_chosen = action

        Qc = Q[chosen]
        Qnc = Q[not_chosen]     
        QA = Q[0]
        QB = Q[1]
        
        probs = softmaxBias(QA, QB, beta, bias)[chosen]
        prob_log += np.log(softmaxBias(QA, QB, beta, bias)[chosen])    
        pA = softmaxBias(QA, QB, beta, bias)[0]
        pB = softmaxBias(QA, QB, beta, bias)[1]
              
        fdbk = row_i.reward
        PE = fdbk - Qc
        
        if fdbk == 0:
            Q_new = Qc + alpha_neg*PE           
        elif fdbk == 1:
            Q_new = Qc + alpha_pos*PE
            
        Q[chosen] = Q_new
                 
        df.loc[i, 'Q_Chosen'] = Qc
        df.loc[i, 'Q_NotChosen'] = Qnc
        df.loc[i, 'Q_Chosen_updated'] = Q_new
        df.loc[i, 'Q_left'] = QA
        df.loc[i, 'Q_right'] = QB
        df.loc[i, 'pA'] = pA
        df.loc[i, 'pB'] = pB
        df.loc[i, 'PE'] = PE             
        
    return df
```

In [ ]:

```
def TwoAlphaBiasForget_like(params,df, model):

    alpha_pos, alpha_neg, beta, bias, forget = params
    
    Q = {0:0.5,1:0.5}
    
    prob_log = 0
    i = 0

    for i, row_i in df.iterrows():
        chosen = row_i.action
        for action in Q.keys():
            if action != chosen:
                not_chosen = action

        Qc = Q[chosen]
        Qnc = Q[not_chosen]
        
        QA = Q[0]
        QB = Q[1]
        
        probs = softmaxBias(QA, QB, beta, bias)[chosen]
        prob_log += np.log(softmaxBias(QA, QB, beta, bias)[chosen])    
        pA = softmaxBias(QA, QB, beta, bias)[0]
        pB = softmaxBias(QA, QB, beta, bias)[1]
    
        fdbk = row_i.reward
        PE = fdbk - Qc
        
        if fdbk == 0:
            Q_new = Qc + alpha_neg*PE
            Q_nc_new = (1-forget) * Qnc
            
        elif fdbk == 1:
            Q_new = Qc + alpha_pos*PE
            Q_nc_new = (1-forget) * Qnc

        Q[chosen] = Q_new
        Q[not_chosen] = Q_nc_new

    prob_log *= -1
    return prob_log

def TwoAlphaBiasForget_return_df(best_ag, best_al, best_b, best_bias, best_forget, df):
        
    alpha_pos = best_ag
    alpha_neg = best_al
    beta = best_b
    bias = best_bias
    forget = best_forget
    
    Q = {0:0.5,1:0.5}
    
    prob_log = 0
    i = 0

    for i, row_i in df.iterrows():
        chosen = row_i.action
        for action in Q.keys():
            if action != chosen:
                not_chosen = action

        Qc = Q[chosen]
        Qnc = Q[not_chosen]
        
        QA = Q[0]
        QB = Q[1]
        
        probs = softmaxBias(QA, QB, beta, bias)[chosen]
        prob_log += np.log(softmaxBias(QA, QB, beta, bias)[chosen])    
        pA = softmaxBias(QA, QB, beta, bias)[0]
        pB = softmaxBias(QA, QB, beta, bias)[1]
              
        fdbk = row_i.reward
        PE = fdbk - Qc
        
        if fdbk == 0:
            Q_new = Qc + alpha_neg*PE
            Q_nc_new = (1-forget) * Qnc
            
        elif fdbk == 1:
            Q_new = Qc + alpha_pos*PE
            Q_nc_new = (1-forget) * Qnc

        Q[chosen] = Q_new
        Q[not_chosen] = Q_nc_new
                 
        df.loc[i, 'Q_Chosen'] = Qc
        df.loc[i, 'Q_NotChosen'] = Qnc
        df.loc[i, 'Q_Chosen_updated'] = Q_new
        df.loc[i, 'Q_NotChosen_updated'] = Q_nc_new
        df.loc[i, 'Q_left'] = QA
        df.loc[i, 'Q_right'] = QB
        df.loc[i, 'pA'] = pA
        df.loc[i, 'pB'] = pB
        df.loc[i, 'PE'] = PE             
        
    return df
```

# Fit models to behavior¶

In [ ]:

```
model_data = mod_data

Q_model = 'one_alpha'
QLearn_like = OneAlpha_like  
bounds_list = [(0.0, 1.0), (0.0, 10.0)]
fit_param_list = ['alpha', 'beta']
num_params = len(fit_param_list)


dfs=[]
for treats in model_data.dose.unique():
    for subj in model_data.subject.unique():
        cdf = model_data[(model_data.dose == treats)&(model_data.subject == subj)]
        cdf.reset_index(inplace=True,drop=True)       

        fit_params = Fit_Function(df=cdf, 
                                  model=Q_model, 
                                  QLearn_like=QLearn_like, 
                                  bounds_list=bounds_list,
                                  fit_param_list = fit_param_list,
                                  num_iters = num_iters)

        ll = fit_params[0]  
        best_a = fit_params[1]
        best_b = fit_params[2]

        bic = GetBIC(ll=ll,nparams=num_params,ntrials=len(cdf))

        mdf = OneAlpha_return_df(best_a, best_b, df=cdf)
        mdf.reset_index(inplace=True,drop=True)  

        mdf['ll'] = ll
        mdf['alpha'] = best_a
        mdf['beta'] = best_b
        mdf['bic'] = bic

        dfs.append(mdf)

oa_res = pd.concat(dfs) 
oa_res.reset_index(inplace=True,drop=True)
oa_res.to_csv(save_path + str(Q_model)  + '_' + str(opt_method) + '.csv',index=False)
oa_res.head()
```

In [ ]:

```
model_data = mod_data

Q_model = 'one_alpha'
QLearn_like = OneAlphaDoubleUpdate_like  
bounds_list = [(0.0, 1.0), (0.0, 10.0)]
fit_param_list = ['alpha', 'beta']
num_params = len(fit_param_list)


dfs=[]
for treats in model_data.dose.unique():
    for subj in model_data.subject.unique():
        cdf = model_data[(model_data.dose == treats)&(model_data.subject == subj)]
        cdf.reset_index(inplace=True,drop=True)       

        fit_params = Fit_Function(df=cdf, 
                                  model=Q_model, 
                                  QLearn_like=QLearn_like, 
                                  bounds_list=bounds_list,
                                  fit_param_list = fit_param_list,
                                  num_iters = num_iters)

        ll = fit_params[0]  
        best_a = fit_params[1]
        best_b = fit_params[2]

        bic = GetBIC(ll=ll,nparams=num_params,ntrials=len(cdf))

        mdf = OneAlphaDoubleUpdate_return_df(best_a, best_b, df=cdf)
        mdf.reset_index(inplace=True,drop=True)  

        mdf['ll'] = ll
        mdf['alpha'] = best_a
        mdf['beta'] = best_b
        mdf['bic'] = bic

        dfs.append(mdf)

oaDU_res = pd.concat(dfs) 
oaDU_res.reset_index(inplace=True,drop=True)
oaDU_res.to_csv(save_path + str(Q_model)  + '_' + str(opt_method) + '.csv',index=False)
oaDU_res.head()
```

In [ ]:

```
model_data = mod_data

Q_model = 'one_alpha_forget'
QLearn_like = OneAlphaForget_like  
bounds_list = [(0.0, 1.0), (0.0, 10.0), (0.0, 1.0)]
fit_param_list = ['alpha', 'beta', 'forget']
num_params = len(fit_param_list)


dfs=[]
for treats in model_data.dose.unique():
    for subj in model_data.subject.unique():
        cdf = model_data[(model_data.dose == treats)&(model_data.subject == subj)]
        cdf.reset_index(inplace=True,drop=True)       

        fit_params = Fit_Function(df=cdf, 
                                  model=Q_model, 
                                  QLearn_like=QLearn_like, 
                                  bounds_list=bounds_list,
                                  fit_param_list = fit_param_list,
                                  num_iters = num_iters)

        ll = fit_params[0]  
        best_a = fit_params[1]
        best_b = fit_params[2]
        best_f = fit_params[3]

        bic = GetBIC(ll=ll,nparams=num_params,ntrials=len(cdf))

        mdf = OneAlphaForget_return_df(best_a, best_b, best_f, df=cdf)
        mdf.reset_index(inplace=True,drop=True)  

        mdf['ll'] = ll
        mdf['alpha'] = best_a
        mdf['beta'] = best_b
        mdf['forget'] = best_f
        mdf['bic'] = bic

        dfs.append(mdf)

oaf_res = pd.concat(dfs) 
oaf_res.reset_index(inplace=True,drop=True)
oaf_res.to_csv(save_path + str(Q_model)  + '_' + str(opt_method) + '.csv',index=False)
oaf_res.head()
```

In [ ]:

```
model_data = mod_data

Q_model = 'one_alpha_bias'
QLearn_like = OneAlphaBias_like  
bounds_list = [(0.0, 1.0), (0.0, 10.0), (-1.0, 1.0)]
fit_param_list = ['alpha', 'beta', 'bias']
num_params = len(fit_param_list)


dfs=[]
for treats in model_data.dose.unique():
    for subj in model_data.subject.unique():
        cdf = model_data[(model_data.dose == treats)&(model_data.subject == subj)]
        cdf.reset_index(inplace=True,drop=True)       

        fit_params = Fit_Function(df=cdf, 
                                  model=Q_model, 
                                  QLearn_like=QLearn_like, 
                                  bounds_list=bounds_list,
                                  fit_param_list = fit_param_list,
                                  num_iters = num_iters)

        ll = fit_params[0]  
        best_a = fit_params[1]
        best_b = fit_params[2]
        best_bias = fit_params[3]

        bic = GetBIC(ll=ll,nparams=num_params,ntrials=len(cdf))

        mdf = OneAlphaBias_return_df(best_a, best_b, best_bias, df=cdf)
        mdf.reset_index(inplace=True,drop=True)  

        mdf['ll'] = ll
        mdf['alpha'] = best_a
        mdf['beta'] = best_b
        mdf['bias'] = best_bias
        mdf['bic'] = bic

        dfs.append(mdf)

oab_res = pd.concat(dfs) 
oab_res.reset_index(inplace=True,drop=True)
oab_res.to_csv(save_path + str(Q_model)  + '_' + str(opt_method) + '.csv',index=False)
oab_res.head()
```

In [ ]:

```
model_data = mod_data

Q_model = 'one_alpha_bias_forget'
QLearn_like = OneAlphaBiasForget_like  
bounds_list = [(0.0, 1.0), (0.0, 10.0), (-1.0, 1.0), (0.0, 1.0)]
fit_param_list = ['alpha', 'beta', 'bias', 'forget']
num_params = len(fit_param_list)


dfs=[]
for treats in model_data.dose.unique():
    for subj in model_data.subject.unique():
        cdf = model_data[(model_data.dose == treats)&(model_data.subject == subj)]
        cdf.reset_index(inplace=True,drop=True)       

        fit_params = Fit_Function(df=cdf, 
                                  model=Q_model, 
                                  QLearn_like=QLearn_like, 
                                  bounds_list=bounds_list,
                                  fit_param_list = fit_param_list,
                                  num_iters = num_iters)

        ll = fit_params[0]  
        best_a = fit_params[1]
        best_b = fit_params[2]
        best_bias = fit_params[3]
        best_f = fit_params[4]

        bic = GetBIC(ll=ll,nparams=num_params,ntrials=len(cdf))

        mdf = OneAlphaBiasForget_return_df(best_a, best_b, best_bias, best_f, df=cdf)
        mdf.reset_index(inplace=True,drop=True)  

        mdf['ll'] = ll
        mdf['alpha'] = best_a
        mdf['beta'] = best_b
        mdf['bias'] = best_bias
        mdf['forget'] = best_f
        mdf['bic'] = bic

        dfs.append(mdf)

oabf_res = pd.concat(dfs) 
oabf_res.reset_index(inplace=True,drop=True)
oabf_res.to_csv(save_path + str(Q_model)  + '_' + str(opt_method) + '.csv',index=False)
oabf_res.head()
```

In [ ]:

```
model_data = mod_data

Q_model = 'two_alpha'
QLearn_like = TwoAlpha_like  
bounds_list = [(0.0, 1.0), (0.0, 1.0), (0.0, 10.0)]
fit_param_list = ['alpha_gain', 'alpha_loss', 'beta']
num_params = len(fit_param_list)


dfs=[]
for treats in model_data.dose.unique():
    for subj in model_data.subject.unique():
        cdf = model_data[(model_data.dose == treats)&(model_data.subject == subj)]
        cdf.reset_index(inplace=True,drop=True)       

        fit_params = Fit_Function(df=cdf, 
                                  model=Q_model, 
                                  QLearn_like=QLearn_like, 
                                  bounds_list=bounds_list,
                                  fit_param_list = fit_param_list,
                                  num_iters = num_iters)

        ll = fit_params[0]  
        best_ag = fit_params[1]
        best_al = fit_params[2]
        best_b = fit_params[3]

        bic = GetBIC(ll=ll,nparams=num_params,ntrials=len(cdf))

        mdf = TwoAlpha_return_df(best_ag, best_al, best_b, df=cdf)
        mdf.reset_index(inplace=True,drop=True)  

        mdf['ll'] = ll
        mdf['alpha_gain'] = best_ag
        mdf['alpha_loss'] = best_al
        mdf['beta'] = best_b
        mdf['bic'] = bic

        dfs.append(mdf)

ta_res = pd.concat(dfs) 
ta_res.reset_index(inplace=True,drop=True)
ta_res.to_csv(save_path + str(Q_model)  + '_' + str(opt_method) + '.csv',index=False)
ta_res.head()
```

In [ ]:

```
model_data = mod_data

Q_model = 'two_alpha_double_update'
QLearn_like = TwoAlphaDoubleUpdate_like  
bounds_list = [(0.0, 1.0), (0.0, 1.0), (0.0, 10.0)]
fit_param_list = ['alpha_gain', 'alpha_loss', 'beta']
num_params = len(fit_param_list)


dfs=[]
for treats in model_data.dose.unique():
    for subj in model_data.subject.unique():
        cdf = model_data[(model_data.dose == treats)&(model_data.subject == subj)]
        cdf.reset_index(inplace=True,drop=True)       

        fit_params = Fit_Function(df=cdf, 
                                  model=Q_model, 
                                  QLearn_like=QLearn_like, 
                                  bounds_list=bounds_list,
                                  fit_param_list = fit_param_list,
                                  num_iters = num_iters)

        ll = fit_params[0]  
        best_ag = fit_params[1]
        best_al = fit_params[2]
        best_b = fit_params[3]

        bic = GetBIC(ll=ll,nparams=num_params,ntrials=len(cdf))

        mdf = TwoAlphaDoubleUpdate_return_df(best_ag, best_al, best_b, df=cdf)
        mdf.reset_index(inplace=True,drop=True)  

        mdf['ll'] = ll
        mdf['alpha_gain'] = best_ag
        mdf['alpha_loss'] = best_al
        mdf['beta'] = best_b
        mdf['bic'] = bic

        dfs.append(mdf)

taDU_res = pd.concat(dfs) 
taDU_res.reset_index(inplace=True,drop=True)
taDU_res.to_csv(save_path + str(Q_model)  + '_' + str(opt_method) + '.csv',index=False)
taDU_res.head()
```

In [ ]:

```
model_data = mod_data

Q_model = 'two_alpha_forget'
QLearn_like = TwoAlphaForget_like  
bounds_list = [(0.0, 1.0), (0.0, 1.0), (0.0, 10.0), (0.0, 1.0)]
fit_param_list = ['alpha_gain', 'alpha_loss', 'beta', 'forget']
num_params = len(fit_param_list)


dfs=[]
for treats in model_data.dose.unique():
    for subj in model_data.subject.unique():
        cdf = model_data[(model_data.dose == treats)&(model_data.subject == subj)]
        cdf.reset_index(inplace=True,drop=True)       

        fit_params = Fit_Function(df=cdf, 
                                  model=Q_model, 
                                  QLearn_like=QLearn_like, 
                                  bounds_list=bounds_list,
                                  fit_param_list = fit_param_list,
                                  num_iters = num_iters)

        ll = fit_params[0]  
        best_ag = fit_params[1]
        best_al = fit_params[2]
        best_b = fit_params[3]
        best_f = fit_params[4]

        bic = GetBIC(ll=ll,nparams=num_params,ntrials=len(cdf))

        mdf = TwoAlphaForget_return_df(best_ag, best_al, best_b, best_f, df=cdf)
        mdf.reset_index(inplace=True,drop=True)  

        mdf['ll'] = ll
        mdf['alpha_gain'] = best_ag
        mdf['alpha_loss'] = best_al
        mdf['beta'] = best_b
        mdf['forget'] = best_f
        mdf['bic'] = bic

        dfs.append(mdf)

taf_res = pd.concat(dfs) 
taf_res.reset_index(inplace=True,drop=True)
taf_res.to_csv(save_path + str(Q_model)  + '_' + str(opt_method) + '.csv',index=False)
taf_res.head()
```

In [ ]:

```
model_data = mod_data

Q_model = 'two_alpha_bias'
QLearn_like = TwoAlphaBias_like  
bounds_list = [(0.0, 1.0), (0.0, 1.0), (0.0, 10.0), (-1.0, 1.0)]
fit_param_list = ['alpha_gain', 'alpha_loss', 'beta', 'bias']
num_params = len(fit_param_list)


dfs=[]
for treats in model_data.dose.unique():
    for subj in model_data.subject.unique():
        cdf = model_data[(model_data.dose == treats)&(model_data.subject == subj)]
        cdf.reset_index(inplace=True,drop=True)       

        fit_params = Fit_Function(df=cdf, 
                                  model=Q_model, 
                                  QLearn_like=QLearn_like, 
                                  bounds_list=bounds_list,
                                  fit_param_list = fit_param_list,
                                  num_iters = num_iters)

        ll = fit_params[0]  
        best_ag = fit_params[1]
        best_al = fit_params[2]
        best_b = fit_params[3]
        best_bias = fit_params[4]

        bic = GetBIC(ll=ll,nparams=num_params,ntrials=len(cdf))

        mdf = TwoAlphaBias_return_df(best_ag, best_al, best_b, best_bias, df=cdf)
        mdf.reset_index(inplace=True,drop=True)  

        mdf['ll'] = ll
        mdf['alpha_gain'] = best_ag
        mdf['alpha_loss'] = best_al
        mdf['beta'] = best_b
        mdf['bias'] = best_bias
        mdf['bic'] = bic

        dfs.append(mdf)

tab_res = pd.concat(dfs) 
tab_res.reset_index(inplace=True,drop=True)
tab_res.to_csv(save_path + str(Q_model)  + '_' + str(opt_method) + '.csv',index=False)
tab_res.head()
```

In [ ]:

```
model_data = mod_data

Q_model = 'two_alpha_bias_forget'
QLearn_like = TwoAlphaBiasForget_like  
bounds_list = [(0.0, 1.0), (0.0, 1.0), (0.0, 10.0), (-1.0, 1.0), (0.0, 1.0)]
fit_param_list = ['alpha_gain', 'alpha_loss', 'beta', 'bias', 'forget']
num_params = len(fit_param_list)


dfs=[]
for treats in model_data.dose.unique():
    for subj in model_data.subject.unique():
        cdf = model_data[(model_data.dose == treats)&(model_data.subject == subj)]
        cdf.reset_index(inplace=True,drop=True)       

        fit_params = Fit_Function(df=cdf, 
                                  model=Q_model, 
                                  QLearn_like=QLearn_like, 
                                  bounds_list=bounds_list,
                                  fit_param_list = fit_param_list,
                                  num_iters = num_iters)

        ll = fit_params[0]  
        best_ag = fit_params[1]
        best_al = fit_params[2]
        best_b = fit_params[3]
        best_bias = fit_params[4]
        best_f = fit_params[5]

        bic = GetBIC(ll=ll,nparams=num_params,ntrials=len(cdf))

        mdf = TwoAlphaBiasForget_return_df(best_ag, best_al, best_b, best_bias, best_f, df=cdf)
        mdf.reset_index(inplace=True,drop=True)  

        mdf['ll'] = ll
        mdf['alpha_gain'] = best_ag
        mdf['alpha_loss'] = best_al
        mdf['beta'] = best_b
        mdf['bias'] = best_bias
        mdf['forget'] = best_f
        mdf['bic'] = bic

        dfs.append(mdf)

tabf_res = pd.concat(dfs) 
tabf_res.reset_index(inplace=True,drop=True)
tabf_res.to_csv(save_path + str(Q_model)  + '_' + str(opt_method) + '.csv',index=False)
tabf_res.head()
```

## Load fitted data¶

In [ ]:

```
save_path = base_dir + 'Processed_data/'

filePath = base_dir + "Analysis/"
all_files = glob.glob(filePath + "/*.csv")

file = []
for filename in all_files:
    df = pd.read_csv(filename, index_col=None, header=0)
    df['filename'] = filename
    file.append(df)
df = pd.concat(file, axis=0, ignore_index=True)

df['model'] = df['filename'].map(lambda x: str(x)[87:-4])
```

In [ ]:

```
print('BIC')

print('----')

print(df.groupby(['model'])['bic'].mean().reset_index().sort_values(by=['bic']))
```

In [ ]:

```
best_model = df[(df.model == 'one_alpha_forget_TNC')]

best_model = best_model.dropna(axis=1, how='all')
best_model.reset_index(inplace=True, drop=True)
```

In [ ]:

```
dfs = []
for subj in raw_data.subject.unique():
    for treats in raw_data.treat.unique():
        sdf = raw_data[(raw_data.subject == subj)&(raw_data.treat == treats)]
        sdf.reset_index(inplace=True,drop=True)
        sdf['trial'] = range(1,len(sdf)+1)
        dfs.append(sdf)
raw_data = pd.concat(dfs)
raw_data.reset_index(inplace=True,drop=True)


dfs = []
for treats in raw_data.treat.unique():
    ddf = raw_data[(raw_data.treat == treats)]
    total_trials = []
    for subj in ddf.subject.unique():
        sdf = ddf[(ddf.subject == subj)] 
        sdf.reset_index(inplace=True,drop=True)
        totat_trials = len(sdf)
        sdf['completed_trials'] = totat_trials
        dfs.append(sdf)            
raw_data = pd.concat(dfs)
raw_data.reset_index(inplace=True,drop=True) 


dfs = []
for treats in raw_data.treat.unique():
    ddf = raw_data[(raw_data.treat == treats)]
    revs = []
    for subj in ddf.subject.unique():
        sdf = ddf[(ddf.subject == subj)] 
        sdf.reset_index(inplace=True,drop=True)
        revs = sdf['SwitchContingency'].cumsum()
        sdf['revs'] = revs.max()
        dfs.append(sdf)            
raw_data = pd.concat(dfs)
raw_data.reset_index(inplace=True,drop=True) 


dfs = []
for treats in raw_data.treat.unique():
    ddf = raw_data[(raw_data.treat == treats)]
    for subj in ddf.subject.unique():
        sdf = ddf[(ddf.subject == subj)] 
        sdf.reset_index(inplace=True,drop=True)    
        revs = sdf.revs.max()
        trials = len(sdf)
        revs100 = revs / trials * 100    
        sdf['revs_per_100'] = revs100
        dfs.append(sdf)            
raw_data = pd.concat(dfs)
raw_data.reset_index(inplace=True,drop=True) 


dfs = []
for treats in raw_data.treat.unique():
    ddf = raw_data[(raw_data.treat == treats)]
    for subj in ddf.subject.unique():
        sdf = ddf[(ddf.subject == subj)] 
        sdf.reset_index(inplace=True,drop=True) 

        repeat_column = []
        repeat = 0
        current_choice = None
        last_choice = None
        
        sdf.reset_index(inplace=True,drop=True)
        for i, row in sdf.iterrows():
            
            if  row.SidePressed == 'L':
                current_choice = 'left'
                
            elif row.SidePressed == 'R':
                current_choice = 'right'
                               
            if current_choice == last_choice:               
                repeat = 1
                
            elif current_choice != last_choice:
                repeat = 0
                
            repeat_column.append(repeat)
 
            last_choice = current_choice

        sdf['repeat'] = repeat_column  
        sdf['repeat_next_trial'] = sdf['repeat'].shift(-1)

        dfs.append(sdf)            
raw_data = pd.concat(dfs)
raw_data.reset_index(inplace=True,drop=True)

raw_data['repeat_next_trial'] = raw_data['repeat_next_trial'].fillna(0)


dfs = []
for treats in raw_data.treat.unique():
    ddf = raw_data[(raw_data.treat == treats)]
    for subj in ddf.subject.unique():
        sdf = ddf[(ddf.subject == subj)] 
        sdf.reset_index(inplace=True,drop=True) 
        
        Tar_Rew = []
        Tar_Loss = []
        NonTar_Rew = []
        NonTar_Loss = []
        Rew = []
        NoRew = []

        T_Rew = 0
        T_Loss = 0
        NT_Rew = 0
        NT_Loss = 0
        rew = 0
        norew = 0

        for i, row in sdf.iterrows():          
            if row.response == 'target' and row.reward == 1:
                T_Rew = 1
                T_Loss = 0
                NT_Rew = 0
                NT_Loss = 0 
                rew = 1
                norew = 0
            elif  row.response == 'target' and row.reward == 0:
                T_Rew = 0
                T_Loss = 1
                NT_Rew = 0
                NT_Loss = 0  
                rew = 0
                norew = 1
            elif row.response == 'non_target' and row.reward == 1:
                T_Rew = 0
                T_Loss = 0
                NT_Rew = 1
                NT_Loss = 0 
                rew = 1
                norew = 0
            elif row.response == 'non_target' and row.reward == 0:
                T_Rew = 0
                T_Loss = 0
                NT_Rew = 0
                NT_Loss = 1 
                rew = 0
                norew = 1
            else:
                T_Rew = 0
                T_Loss = 0
                NT_Rew = 0
                NT_Loss = 0  
                rew = 0
                norew = 0
                
            Tar_Rew.append(T_Rew)
            Tar_Loss.append(T_Loss)
            NonTar_Rew.append(NT_Rew)
            NonTar_Loss.append(NT_Loss)  
            Rew.append(rew)
            NoRew.append(norew)  
        sdf['T_Rew'] = Tar_Rew
        sdf['T_Loss'] = Tar_Loss 
        sdf['NT_Rew'] = NonTar_Rew
        sdf['NT_Loss'] = NonTar_Loss  
        sdf['Rew'] = Rew
        sdf['NoRew'] = NoRew  
        dfs.append(sdf)            
raw_data = pd.concat(dfs)
raw_data.reset_index(inplace=True,drop=True) 


dfs = []
for treats in raw_data.treat.unique():
    ddf = raw_data[(raw_data.treat == treats)]
    for subj in ddf.subject.unique():
        sdf = ddf[(ddf.subject == subj)] 
        sdf.reset_index(inplace=True,drop=True) 
        
        TargetWS = []
        TargetLS = []
        NonTargetWS = []
        NonTargetLS = []
        WinStay = []
        LoseShift = []
        
        TWS = 0
        TLS = 0
        NTWS = 0
        NTLS = 0
        WS = 0
        LS = 0
        
        for i, row in sdf.iterrows():          

            if row.repeat_next_trial == 1 and row.T_Rew == 1:
                TWS = 1
                TLS = 0
                NTWS = 0
                NTLS = 0
                WS = 1
                LS = 0

            elif row.repeat_next_trial == 0 and row.T_Loss == 1:
                TWS = 0
                TLS = 1
                NTWS = 0
                NTLS = 0
                WS = 0
                LS = 1
                
            elif row.repeat_next_trial == 1 and row.NT_Rew == 1:
                TWS = 0
                TLS = 0
                NTWS = 1
                NTLS = 0
                WS = 1
                LS = 0
                
            elif row.repeat_next_trial == 0 and row.NT_Loss == 1:
                TWS = 0
                TLS = 0
                NTWS = 0
                NTLS = 1
                WS = 0
                LS = 1
                
            else: 
                TWS = 0
                TLS = 0
                NTWS = 0
                NTLS = 0
                WS = 0
                LS = 0
              
            TargetWS.append(TWS)
            TargetLS.append(TLS)
            NonTargetWS.append(NTWS)
            NonTargetLS.append(NTLS)
            
            WinStay.append(WS)
            LoseShift.append(LS)
            
        sdf['TargetWinStay'] = TargetWS
        sdf['TargetLoseShift'] = TargetLS
        sdf['NonTargetWinStay'] = NonTargetWS
        sdf['NonTargetLoseShift'] = NonTargetLS
  
        sdf['WinStay'] = WinStay
        sdf['LoseShift'] = LoseShift
  
        dfs.append(sdf)     
raw_data = pd.concat(dfs)
raw_data.reset_index(inplace=True,drop=True)  


dfs = []
for treats in raw_data.treat.unique():
    ddf = raw_data[(raw_data.treat == treats)]
    for subj in ddf.subject.unique():
        sdf = ddf[(ddf.subject == subj)] 
        sdf.reset_index(inplace=True,drop=True) 
        
        sdf['TWS'] = sdf['TargetWinStay'].sum() /  sdf['T_Rew'].sum()
        sdf['TLS'] = sdf['TargetLoseShift'].sum() /  sdf['T_Loss'].sum()
        sdf['NTLS'] = sdf['NonTargetLoseShift'].sum() /  sdf['NT_Loss'].sum()
        sdf['NTWS'] = sdf['NonTargetWinStay'].sum() /  sdf['NT_Rew'].sum()  
        
        sdf['WS'] = sdf['WinStay'].sum() /  sdf['Rew'].sum()
        sdf['LS'] = sdf['LoseShift'].sum() /  sdf['NoRew'].sum()  
        
        dfs.append(sdf)
raw_data = pd.concat(dfs)
raw_data.reset_index(inplace=True,drop=True)
```

# Posterior Predictive Check¶

In [ ]:

```
def RandomStart():
    start = np.random.randint(2, size=1)
    if start == 1:
        rew_p = [0.2, 0.8]      
    else:
        rew_p = [0.8, 0.2]
    return rew_p

def RewardDeliveryAccuracy(action, reward_prob):
    rew_p = reward_prob[action]
    norew_p = 1-rew_p    
    reward = np.random.choice(a=[0,1],p=[norew_p,rew_p])
    accuracy = 0 if rew_p < norew_p else 1
    return reward, accuracy

def softmax(QA, QB, beta):
    """Compute softmax probabilities for all actions."""
    Q = np.array((QA, QB))
    return np.exp(Q * beta) / np.exp(Q * beta).sum()

def reversal_trigger(rew_p, corr_ct, accuracy, stage):   

    if corr_ct == 7 and accuracy == 1:
        rev = 1 
        rew_p.reverse()
        corr_ct = 0
        stage = stage + 1
        
    elif corr_ct <7 and accuracy == 1:
        rev = 0   
        corr_ct += 1
        stage = stage
        
    elif accuracy == 0:
        rev = 0      
        corr_ct = 0
        stage = stage
        
    return rew_p, corr_ct, rev, stage
```

In [ ]:

```
def simulated_WSLS(simulated_df):    

    sim_data = simulated_df

    
    dfs = []
    for treats in sim_data.treat.unique():
        ddf = sim_data[(sim_data.treat == treats)]
        for subj in ddf.subject.unique():
            sdf = ddf[(ddf.subject == subj)] 
            sdf.reset_index(inplace=True,drop=True) 
            revs = sdf['reversal'].cumsum()
            sdf['revs'] = revs.max()
            dfs.append(sdf)            
    sim_data = pd.concat(dfs)
    sim_data.reset_index(inplace=True,drop=True) 

    dfs = []
    for treats in sim_data.treat.unique():
        ddf = sim_data[(sim_data.treat == treats)]
        for subj in ddf.subject.unique():
            sdf = ddf[(ddf.subject == subj)] 
            sdf.reset_index(inplace=True,drop=True) 
            revs = sdf.revs.max()
            trials = len(sdf)
            revs100 = revs / trials * 100    
            sdf['revs_per_100'] = revs100
            dfs.append(sdf)            
    sim_data = pd.concat(dfs)
    sim_data.reset_index(inplace=True,drop=True) 

    
    dfs = []
    for treats in sim_data.treat.unique():
        ddf = sim_data[(sim_data.treat == treats)]
        for subj in ddf.subject.unique():
            sdf = ddf[(ddf.subject == subj)] 
            sdf.reset_index(inplace=True,drop=True) 

            repeat_column = []
            repeat = 0
            current_choice = None
            last_choice = None

            sdf.reset_index(inplace=True,drop=True)
            for i, row in sdf.iterrows():

                if  row.action == 0:
                    current_choice = 'left'

                elif row.action == 1:
                    current_choice = 'right'

                if current_choice == last_choice:               
                    repeat = 1

                elif current_choice != last_choice:
                    repeat = 0

                repeat_column.append(repeat)

                last_choice = current_choice

            sdf['repeat'] = repeat_column  
            sdf['repeat_next_trial'] = sdf['repeat'].shift(-1)

            dfs.append(sdf)            
    sim_data = pd.concat(dfs)
    sim_data.reset_index(inplace=True,drop=True)

    sim_data['repeat_next_trial'] = sim_data['repeat_next_trial'].fillna(0)


    dfs = []
    for treats in sim_data.treat.unique():
        ddf = sim_data[(sim_data.treat == treats)]
        for subj in ddf.subject.unique():
            sdf = ddf[(ddf.subject == subj)] 
            sdf.reset_index(inplace=True,drop=True) 

            Tar_Rew = []
            Tar_Loss = []
            NonTar_Rew = []
            NonTar_Loss = []
            Rew = []
            NoRew = []

            T_Rew = 0
            T_Loss = 0
            NT_Rew = 0
            NT_Loss = 0
            rew = 0
            norew = 0

            for i, row in sdf.iterrows():          
                if row.accuracy == 1 and row.reward == 1:
                    T_Rew = 1
                    T_Loss = 0
                    NT_Rew = 0
                    NT_Loss = 0 
                    rew = 1
                    norew = 0
                elif  row.accuracy == 1 and row.reward == 0:
                    T_Rew = 0
                    T_Loss = 1
                    NT_Rew = 0
                    NT_Loss = 0  
                    rew = 0
                    norew = 1
                elif row.accuracy == 0 and row.reward == 1:
                    T_Rew = 0
                    T_Loss = 0
                    NT_Rew = 1
                    NT_Loss = 0 
                    rew = 1
                    norew = 0
                elif row.accuracy == 0 and row.reward == 0:
                    T_Rew = 0
                    T_Loss = 0
                    NT_Rew = 0
                    NT_Loss = 1 
                    rew = 0
                    norew = 1
                else:
                    T_Rew = 0
                    T_Loss = 0
                    NT_Rew = 0
                    NT_Loss = 0  
                    rew = 0
                    norew = 0

                Tar_Rew.append(T_Rew)
                Tar_Loss.append(T_Loss)
                NonTar_Rew.append(NT_Rew)
                NonTar_Loss.append(NT_Loss)  
                Rew.append(rew)
                NoRew.append(norew)  
            sdf['T_Rew'] = Tar_Rew
            sdf['T_Loss'] = Tar_Loss 
            sdf['NT_Rew'] = NonTar_Rew
            sdf['NT_Loss'] = NonTar_Loss  
            sdf['Rew'] = Rew
            sdf['NoRew'] = NoRew  
            dfs.append(sdf)            
    sim_data = pd.concat(dfs)
    sim_data.reset_index(inplace=True,drop=True) 

    dfs = []
    for treats in sim_data.treat.unique():
        ddf = sim_data[(sim_data.treat == treats)]
        for subj in ddf.subject.unique():
            sdf = ddf[(ddf.subject == subj)] 
            sdf.reset_index(inplace=True,drop=True) 

            TargetWS = []
            TargetLS = []
            NonTargetWS = []
            NonTargetLS = []
            WinStay = []
            LoseShift = []

            TWS = 0
            TLS = 0
            NTWS = 0
            NTLS = 0
            WS = 0
            LS = 0

            for i, row in sdf.iterrows():          

                if row.repeat_next_trial == 1 and row.accuracy == 1 and row.reward == 1:
                    TWS = 1
                    TLS = 0
                    NTWS = 0
                    NTLS = 0
                    WS = 1
                    LS = 0

                elif row.repeat_next_trial == 0 and row.accuracy == 1 and row.reward == 0:
                    TWS = 0
                    TLS = 1
                    NTWS = 0
                    NTLS = 0
                    WS = 0
                    LS = 1

                elif row.repeat_next_trial == 1 and row.accuracy == 0 and row.reward == 1:
                    TWS = 0
                    TLS = 0
                    NTWS = 1
                    NTLS = 0
                    WS = 1
                    LS = 0

                elif row.repeat_next_trial == 0 and row.accuracy == 0 and row.reward == 0:
                    TWS = 0
                    TLS = 0
                    NTWS = 0
                    NTLS = 1
                    WS = 0
                    LS = 1

                else: 
                    TWS = 0
                    TLS = 0
                    NTWS = 0
                    NTLS = 0
                    WS = 0
                    LS = 0

                TargetWS.append(TWS)
                TargetLS.append(TLS)
                NonTargetWS.append(NTWS)
                NonTargetLS.append(NTLS)

                WinStay.append(WS)
                LoseShift.append(LS)

            sdf['TargetWinStay'] = TargetWS
            sdf['TargetLoseShift'] = TargetLS
            sdf['NonTargetWinStay'] = NonTargetWS
            sdf['NonTargetLoseShift'] = NonTargetLS

            sdf['WinStay'] = WinStay
            sdf['LoseShift'] = LoseShift

            dfs.append(sdf)     
    sim_data = pd.concat(dfs)
    sim_data.reset_index(inplace=True,drop=True)  


    dfs = []
    for treats in sim_data.treat.unique():
        ddf = sim_data[(sim_data.treat == treats)]
        for subj in ddf.subject.unique():
            sdf = ddf[(ddf.subject == subj)] 
            sdf.reset_index(inplace=True,drop=True) 

            sdf['TWS'] = sdf['TargetWinStay'].sum() /  sdf['T_Rew'].sum()
            sdf['TLS'] = sdf['TargetLoseShift'].sum() /  sdf['T_Loss'].sum()
            sdf['NTLS'] = sdf['NonTargetLoseShift'].sum() /  sdf['NT_Loss'].sum()
            sdf['NTWS'] = sdf['NonTargetWinStay'].sum() /  sdf['NT_Rew'].sum()  

            sdf['WS'] = sdf['WinStay'].sum() /  sdf['Rew'].sum()
            sdf['LS'] = sdf['LoseShift'].sum() /  sdf['NoRew'].sum()  

            dfs.append(sdf)
    sim_data = pd.concat(dfs)
    sim_data.reset_index(inplace=True,drop=True)

    return sim_data
```

In [ ]:

```
def OneAlphaForget_sim(params,ntrials):
    
    alpha, beta, forget = params  
       
    rew_p = RandomStart()
        
    Q = {0:0.5, 1:0.5}  

    d = {}
    i=0
    
    stage = 1
    corr_ct = 0
    
    for i in range(ntrials):
        trial_d = {}        
        trial_d['trial'] = i+1
        
        p = softmax(Q[0], Q[1], beta)  
        p0, p1 = p[0], p[1]
        
        chosen = np.random.choice(a=[0,1],p=p)
        not_chosen = 1 - chosen    

        Qc = Q[chosen]
        Qnc = Q[not_chosen]
        
        reward, accuracy = RewardDeliveryAccuracy(chosen, rew_p)
        
        trial_d['action'] = chosen
        trial_d['reward'] = reward
        trial_d['accuracy'] = accuracy
                        
        fdbk = reward
        PE = fdbk - Qc

        Q_new = Qc + alpha * PE
        Q_nc_new = (1-forget) * Qnc
        
        Q[chosen] = Q_new
        Q[not_chosen] = Q_nc_new           
            
        rew_p, corr_ct, rev, stage = reversal_trigger(rew_p, corr_ct, accuracy, stage)  

        trial_d['reversal'] = rev
        trial_d['stage'] = stage
        
        trial_d['Qc'] = Qc
        trial_d['Qnc'] = Qnc
         
        trial_d['alpha'] = alpha
        trial_d['beta'] = beta
        trial_d['forget'] = forget
        
        d[i] = trial_d

    out = pd.DataFrame.from_dict(d,orient='index')
    out.reset_index(inplace=True,drop=True)
    return out
```

In [ ]:

```
data_summary_for_sim = raw_data[(raw_data.model=='one_alpha_forget')].groupby(['treat', 'sex', 'subject'])['revs_per_100', 
                                                                                                           'completed_trials',
                                                                                                           'TWS', 'TLS', 'NTLS', 'NTWS',
                                                                                                           'alpha', 'beta', 'forget'].mean().reset_index()
```

In [ ]:

```
dfs = []
for treats in data_summary_for_sim.treat.unique():
    curr_treat = data_summary_for_sim[(data_summary_for_sim.treat==treats)]
    
    for subj in curr_treat.subject.unique():
        single_subj = curr_treat[(curr_treat.subject == subj)]
        single_subj.reset_index(inplace=True,drop=True)
        
        trial_cnt = single_subj.completed_trials[0].astype(int)
        subj_id = single_subj.subject[0]
        treat_id = single_subj.treat[0]
        
        a = single_subj.alpha[0]
        b = single_subj.beta[0]
        f = single_subj.forget[0]

        curr_params = [a, b, f]

        curr_alpha, curr_beta, curr_forget = curr_params
        
        sdf = OneAlphaForget_sim(params=curr_params,ntrials=trial_cnt) # mean number of trials completed
        
        sdf['treat'] = treat_id
        sdf['subject'] = subj_id
        sdf['completed_trials'] = trial_cnt
        
        sdf['alpha'] = curr_alpha
        sdf['beta'] = curr_beta
        sdf['forget'] = curr_forget

        sdf = sdf[['treat','subject', 'completed_trials', 'trial','action','accuracy','reward', 'reversal', 'alpha', 'beta', 'forget']]
        
        sdf.reset_index(inplace=True,drop=True)
        dfs.append(sdf)   
        
oaf_sim = pd.concat(dfs)
oaf_sim.reset_index(inplace=True,drop=True)
```

In [ ]:

```
oaf_sim_out = simulated_WSLS(oaf_sim)

oaf_sim_out = oaf_sim_out.groupby(['treat', 'subject'])['revs_per_100', 
                                                        'TWS', 'TLS', 'NTLS', 'NTWS', 
                                                        'alpha', 'beta', 'forget'].mean().reset_index()

data_summary_for_sim['group'] = 'rat'
oaf_sim_out['group'] = 'model'

actual_vs_simulated = pd.concat([data_summary_for_sim, oaf_sim_out],axis=0)
```

In [ ]:

```
#actual_vs_simulated.to_csv(save_path+'rat_oaf_PPC.csv')
```

## Parameter Recovery¶

In [ ]:

```
Q_model = 'one_alpha_forget'
QLearn_like = OneAlphaForget_like  
bounds_list = [(0.0, 1.0), (0.0, 10.0), (0.0, 1.0)]
fit_param_list = ['alpha', 'beta', 'forget']
num_params = len(fit_param_list)


run_ct = 0
results = {}

for run in range(1,31):
        
    curr_alpha = np.random.uniform(low=0.001, high=0.999)
    
    if curr_alpha < 0.5:    
        curr_beta = np.random.uniform(low=5.001, high=9.999)
        curr_forget = np.random.uniform(low=0.001, high=0.499)
        
    elif curr_alpha >= 0.5:    
        curr_beta = np.random.uniform(low=0.001, high=4.999)
        curr_forget = np.random.uniform(low=0.4999, high=0.999)
    
    curr_params = [curr_alpha, curr_beta, curr_forget]
        
    trial_num = int(np.random.randint(250, high=300, size=1))
    
    out = OneAlphaForget_sim(params=curr_params,ntrials = trial_num) # mean number of trials completed    
    out = out[['trial', 'action', 'reward']]

    out['subject'] = run_ct
    
    fit_params = Fit_Function(df=out, 
                              model=Q_model, 
                              QLearn_like=QLearn_like, 
                              bounds_list=bounds_list,
                              fit_param_list = fit_param_list,
                              num_iters = num_iters, 
                              opt_method = opt_method)
    
                
    ll = fit_params[0]  
    fit_alpha = fit_params[1]
    fit_beta = fit_params[2]
    fit_forget = fit_params[3]

    results[run_ct] = {'run':run,
                       'll':ll,
                       'actual_alpha':curr_alpha,'fit_alpha':fit_alpha,
                       'actual_beta':curr_beta,'fit_beta':fit_beta,
                       'actual_forget':curr_forget,'fit_forget':fit_forget}
    
    print(run_ct)
    run_ct = run_ct + 1

oaf_pcheck = pd.DataFrame.from_dict(results,orient='index')
```

In [ ]:

```
#oaf_pcheck.to_csv(save_path + 'oaf_pcheck_'+str(opt_method)+'.csv')
```

In [ ]:

```

```
