# Supplementary material for: Identification of conserved frontal neurophysiological markers of cognitive flexibility in humans and rats
Source: Commun Biol. 2025 Aug 23;8:1268. doi: 10.1038/s42003-025-08729-x (PMC12375130; doi:10.1038/s42003-025-08729-x)
Supplement: Supplementary file 7 — Python code for Rodent data analysis [file 42003_2025_8729_MOESM7_ESM.html]

Final\_Rodent\_Analysis


In [ ]:

```
# load libraries needed for analysis
import os

os.environ['KMP_DUPLICATE_LIB_OK']='True'
os.environ['R_HOME']='C:/Users/samba/anaconda3/envs/data_analysis/Lib/R'
os.environ['R_USER']='C:/Users/samba/anaconda3/envs/data_analysis/Lib/R'

import numpy as np
import pandas as pd
import scipy as sp
import seaborn as sns
import glob as glob
from matplotlib import pyplot as plt
import getpass
import math
import re
import warnings

sns.set_style('darkgrid')
sns.color_palette('deep')
```

In [ ]:

```
filePath = "C:/Users/samba/Dropbox/Work Folder/Python Scripts/PRL/UH3_Project_Dec2022_Analysis/Rats_UH3_modafinil/behavior_with_EEG/" 
save_path = "C:/Users/samba/Dropbox/Work Folder/Python Scripts/PRL/UH3_Project_Dec2022_Analysis/Submission 2/Rat/Processed_data/" 

model = 'oaf'

all_files = glob.glob(filePath + model + "/*.csv")

# import all csv files in target dir and concat to df
file = []
for filename in all_files:
    df = pd.read_csv(filename, index_col=None, header=0)
    df['filename'] = filename
    file.append(df)
df = pd.concat(file, axis=0, ignore_index=True)
```

In [ ]:

```
data = df

data = data[(data.subject != 35)]
data.reset_index(inplace=True, drop=True)
```

In [ ]:

```
data['TR'] = data.response_type.replace({'TR':1, 'TNR':0, 'NTNR':0, 'NTR':0})
data['TNR'] = data.response_type.replace({'TR':0, 'TNR':1, 'NTNR':0, 'NTR':0})
data['NTNR'] = data.response_type.replace({'TR':0, 'TNR':0, 'NTNR':1, 'NTR':0})
data['NTR'] = data.response_type.replace({'TR':0, 'TNR':0, 'NTNR':0, 'NTR':1})

data['ERP_early'] = data.loc[:, 'T60':'T160'].mean(axis=1)
data['ERP_late'] = data.loc[:, 'T185':'T305'].mean(axis=1)

data['ERP_diff'] = data['ERP_early'] - data['ERP_late']
```

In [ ]:

```
def makePlots(input_data1, input_data2, input_data3, input_data4, title, measure1, measure2, measure3, measure4, fig_name):
    
    plt.rcParams.update({'font.size': 20})
    
    dataset1 = input_data1
    dataset2 = input_data2
    dataset3 = input_data3
    dataset4 = input_data4
    
    rat_plot1 = []
    for subj in dataset1.subject.unique(): 
        sdf1 = dataset1[(dataset1.subject == subj)]
        rat_id = pd.DataFrame(sdf1.subject.unique())    
        p1 = sdf1.loc[:,'TNeg100':'T395'].mean(axis=0) 
        plot1 = pd.DataFrame(p1).T       
        plot1_and_ID = pd.concat([rat_id, plot1], axis=1)
        rat_plot1.append(plot1_and_ID)

    TR_plot = pd.concat(rat_plot1)
    TR_plot.reset_index(inplace=True,drop=True)
    
    rat_plot2 = []
    for subj in dataset2.subject.unique(): 
        sdf2 = dataset2[(dataset2.subject == subj)]
        rat_id = pd.DataFrame(sdf2.subject.unique()) 
        p2 = sdf2.loc[:,'TNeg100':'T395'].mean(axis=0)
        plot2 = pd.DataFrame(p2).T  
        plot2_and_ID = pd.concat([rat_id, plot2], axis=1)
        rat_plot2.append(plot2_and_ID)

    TNR_plot = pd.concat(rat_plot2)
    TNR_plot.reset_index(inplace=True,drop=True)
   
    rat_plot3 = []
    for subj in dataset3.subject.unique(): 
        sdf3 = dataset3[(dataset3.subject == subj)]
        rat_id = pd.DataFrame(sdf3.subject.unique())
        p3 = sdf3.loc[:,'TNeg100':'T395'].mean(axis=0)
        plot3 = pd.DataFrame(p3).T  
        plot3_and_ID = pd.concat([rat_id, plot3], axis=1)
        rat_plot3.append(plot3_and_ID)

    NTNR_plot = pd.concat(rat_plot3)
    NTNR_plot.reset_index(inplace=True,drop=True)

    rat_plot4 = []
    for subj in dataset4.subject.unique(): 
        sdf4 = dataset4[(dataset4.subject == subj)]
        rat_id = pd.DataFrame(sdf4.subject.unique())
        p4 = sdf4.loc[:,'TNeg100':'T395'].mean(axis=0)
        plot4 = pd.DataFrame(p4).T       
        plot4_and_ID = pd.concat([rat_id, plot4], axis=1)
        rat_plot4.append(plot4_and_ID)

    NTR_plot = pd.concat(rat_plot4)
    NTR_plot.reset_index(inplace=True,drop=True)
       
    TR_error = np.std(TR_plot.loc[:, 'TNeg100':'T395'],axis=0)/np.sqrt(len(TR_plot)) 
    TNR_error = np.std(TNR_plot.loc[:, 'TNeg100':'T395'],axis=0)/np.sqrt(len(TNR_plot)) 
    NTNR_error = np.std(NTNR_plot.loc[:, 'TNeg100':'T395'],axis=0)/np.sqrt(len(NTNR_plot)) 
    NTR_error = np.std(NTR_plot.loc[:, 'TNeg100':'T395'],axis=0)/np.sqrt(len(NTR_plot))     
    

    T_diff = np.subtract(TR_plot.loc[:, 'TNeg100':'T395'],TNR_plot.loc[:, 'TNeg100':'T395'])
    NT_diff = np.subtract(NTR_plot.loc[:, 'TNeg100':'T395'],NTNR_plot.loc[:, 'TNeg100':'T395'])

    T_diff_error = np.std(T_diff.loc[:, 'TNeg100':'T395'],axis=0)/np.sqrt(len(T_diff))   
    NT_diff_error = np.std(NT_diff.loc[:, 'TNeg100':'T395'],axis=0)/np.sqrt(len(NT_diff))   
     
    
    axis1 = TR_plot.columns[1:]
    bins = len(np.mean(TR_plot.loc[:, 'TNeg100':'T395'],axis=0))
    axis1 = np.linspace(-0.1,0.4, num=bins)

    fig = plt.figure(figsize=(8, 12), dpi=600)
    ax1 = fig.add_subplot(311)

    ax1.set_facecolor('white')

    ax1.spines["top"].set_visible(False)
    ax1.spines["right"].set_visible(False)
    ax1.spines['left'].set_color('black')
    ax1.spines['bottom'].set_color('black')
    ax1.spines['left'].set_linewidth(1)
    ax1.spines['bottom'].set_linewidth(1)

    ax1.set_title(title)
    p1, = ax1.plot(axis1, np.mean(TR_plot.loc[:, 'TNeg100':'T395'],axis=0), linewidth=2, color='blue', label=measure1)
    p2, = ax1.plot(axis1, np.mean(TNR_plot.loc[:, 'TNeg100':'T395'],axis=0), linewidth=2, color='red', label=measure2)
    p3, = ax1.plot(axis1, np.mean(NTNR_plot.loc[:, 'TNeg100':'T395'],axis=0), linewidth=2, linestyle='--', color='red', label=measure3)
    p4, = ax1.plot(axis1, np.mean(NTR_plot.loc[:, 'TNeg100':'T395'],axis=0), linewidth=2, linestyle='--', color='blue', label=measure4)

    ax1.axvline(x=0, linewidth=3, linestyle='--', color='slategray', alpha=0.5)   
    ax1.axhline(y=0, linewidth=3, linestyle='--', color='slategray', alpha=0.5)   
    ax1.set_ylabel('activity (uV)')
    ax1.legend(handles=[p1, p2, p3, p4], loc='upper right')
    ax1.set_ylim(-60, 60)
      
    ax2 = fig.add_subplot(312)   
    
    ax2.set_facecolor('white')
    ax2.spines["top"].set_visible(False)
    ax2.spines["right"].set_visible(False)
    ax2.spines['left'].set_color('black')
    ax2.spines['bottom'].set_color('black')
    ax2.spines['left'].set_linewidth(1)
    ax2.spines['bottom'].set_linewidth(1)
           
    axis2 = axis1
    p1, = ax2.plot(axis2, np.mean(T_diff.loc[:, 'TNeg100':'T395'],axis=0), linewidth=2, color='black', label='T diff')
    p2, = ax2.plot(axis2, np.mean(NT_diff.loc[:, 'TNeg100':'T395'],axis=0), linewidth=2, linestyle='--', color='black', label='NT diff')
     
    ax2.axvline(x=0, linewidth=3, linestyle='--', color='slategray', alpha=0.5)   
    ax2.axhline(y=0, linewidth=3, linestyle='--', color='slategray', alpha=0.5)     
    ax2.set_ylabel('activity (uV)')
    ax2.legend(handles=[p1, p2], loc='upper right')
    ax2.set_xlabel('time surrounding tone (s)')
    ax2.set_ylim(-60, 60)   
                       
    xticks = np.arange(-0.1, 0.41, 0.1)
    ax1.set_xticks(xticks)
    ax2.set_xticks(xticks)

    yticks = np.arange(-60, 61, 20)  
    ax1.set_yticks(yticks)
    ax2.set_yticks(yticks)

    plt.tight_layout()  
    #plt.savefig(save_path + fig_name + '.svg', transparent=False)

    return fig
```

In [ ]:

```
plot_data = data[(data.chan=='ACC_LFP')&(data.treatment=='Day 21')]

Fig1 = makePlots(plot_data[(plot_data.TR == 1)], 
                 plot_data[(plot_data.TNR == 1)],
                 plot_data[(plot_data.NTNR == 1)], 
                 plot_data[(plot_data.NTR == 1)], 'Day 21', 'TR', 'TNR', 'NTNR', 'NTR', 'Fig 4 - Day 21 plot')
```

In [ ]:

```
def GLM_one_predictor(input_data, predictors):
        
    import statsmodels.api as sm
    import statsmodels.formula.api as smf

    activity = input_data.loc[:, 'TNeg100':'T395']

    intercept = []
    coef1 = []
    pvalues1 = []
    tvalues1 = []
    std1 = []

    for g in activity:

        timebin = activity[g]
        input_data['bin'] = timebin

        md = smf.glm("bin ~ PE", input_data, family=sm.families.Gaussian())
        mdf = md.fit()

        intercept.append(mdf.params['Intercept'])
        coef1.append(mdf.params[predictors[0]])
        pvalues1.append(mdf.pvalues[predictors[0]])
        tvalues1.append(mdf.tvalues[predictors[0]])
        std1.append(mdf.bse[predictors[0]])
      
    return intercept, coef1, std1, pvalues1
```

In [ ]:

```
def plot_one_betas(betas, pvals_corr, plot_pvals, adjust):
    
    betas = np.array(betas)
    pvals_corr = np.array(pvals_corr)
    
    bins = len(betas)

    axis1 = np.linspace(-0.1,0.4, num=bins)
   
    import matplotlib.ticker as ticker
    parameters = {'axes.labelsize': 25, 'axes.titlesize': 35, 'xtick.labelsize': 20, 'ytick.labelsize': 20}
    plt.rcParams.update(parameters)
    
    fig = plt.figure(figsize=(8, 12), dpi=600)
    ax0 = fig.add_subplot(311)

    ax0.set_facecolor('white')

    ax0.spines["top"].set_visible(False)
    ax0.spines["right"].set_visible(False)

    ax0.spines['left'].set_color('black')
    ax0.spines['bottom'].set_color('black')
    ax0.spines['left'].set_linewidth(1)
    ax0.spines['bottom'].set_linewidth(1)

    p1, = ax0.plot(axis1, betas, linewidth=2, color='blue')

    if plot_pvals == 'yes':
        
        n_pvals = pvals_corr.shape
        max_y1 = betas.max() + adjust

        for i in pvals_corr:
            t = np.linspace(-0.1,0.4, num=bins)
            t05 = t[pvals_corr < 0.05]
            ax0.plot(t05, np.ones(t05.shape)*max_y1, '.', color='b', markersize=6)
    else:
        pass    
        
    ax0.axvline(x=0, linewidth=3, linestyle='--', color='slategray', alpha=0.5)   
    ax0.axhline(y=0, linewidth=3, linestyle='--', color='slategray', alpha=0.5)   
    
    xticks = np.arange(-0.1, 0.41, 0.1)
    yticks = np.arange(-20, 41, 20)  
    
    ax0.set_xticks(xticks)  
    ax0.set_yticks(yticks)   

    ax0.set_ylabel('regression coefficient')
    ax0.legend(handles=[p1], loc='upper right')
    ax0.set_xlabel('time surrounding tone (s)')
    
    #plt.savefig(save_path + 'Rat_Fig3B.svg', transparent=False)
    
    plt.tight_layout()             

    
def predictActivity (PE, model_coefficient, model_intercept):
    
    """
    # function takes a PE value, the model coefficient and the intercept 
    value and returns the predicted GCaMP signal
    # https://datagy.io/python-sklearn-linear-regression/
    """   
    return (PE * model_coefficient) + model_intercept
```

In [ ]:

```
ACC_D21 = data[(data.chan=='ACC_LFP')&(data.treatment=='Day 21')]
```

In [ ]:

```
ACC_D21_regression = ACC_D21
ACC_D21_regression.reward.replace({1:1, 0:-1},inplace=True)
```

In [ ]:

```
PE_intercept, PE_coef, PE_sem, PE_pvalues = GLM_one_predictor(ACC_D21_regression, predictors=['PE'])
```

In [ ]:

```
Fig3B = plot_one_betas(PE_coef,
               PE_pvalues,
               plot_pvals='no', 
               adjust=10)
```

In [ ]:

```
predicted_60 = []
predicted_30 = []
predicted_0 = []
predicted_minus30 = []
predicted_minus60 = []

for x, y in zip(PE_coef, PE_intercept):
                predicted_60.append(predictActivity(0.6, x, y))
        
for x, y in zip(PE_coef, PE_intercept):
                predicted_30.append(predictActivity(0.3, x, y))
        
for x, y in zip(PE_coef, PE_intercept):
                predicted_0.append(predictActivity(0, x, y))
        
for x, y in zip(PE_coef, PE_intercept):
                predicted_minus30.append(predictActivity(-0.3, x, y))
        
for x, y in zip(PE_coef, PE_intercept):
                predicted_minus60.append(predictActivity(-0.6, x, y))
```

In [ ]:

```
bins = len(PE_coef)
axis1 = np.linspace(-0.1,0.4, num=bins)


fig = plt.figure(figsize=(8, 12), dpi=600)
ax0 = fig.add_subplot(311)

ax0.set_facecolor('white')

ax0.spines["top"].set_visible(False)
ax0.spines["right"].set_visible(False)

ax0.spines['left'].set_color('black')
ax0.spines['bottom'].set_color('black')
ax0.spines['left'].set_linewidth(1)
ax0.spines['bottom'].set_linewidth(1)

p1, = ax0.plot(axis1, predicted_60, linewidth=2, color='blue', label='PE, 0.6')
p2, = ax0.plot(axis1, predicted_30, linewidth=2, color='blue', linestyle='--', label='PE, 0.3')

p4, = ax0.plot(axis1, predicted_minus30, linewidth=2, color='red', linestyle='--', label='PE, -0.3')
p5, = ax0.plot(axis1, predicted_minus60, linewidth=2, color='red', label='PE, -0.6')

ax0.axvline(x=0, linewidth=3, linestyle='--', color='slategray', alpha=0.5)   
ax0.axhline(y=0, linewidth=3, linestyle='--', color='slategray', alpha=0.5)   
ax0.legend(handles=[p1, p2, p4, p5], loc='upper right')

ax0.set_ylabel('Predicted ACC LFP activity')
ax0.set_xlabel('time surrounding tone (s)')

xticks = np.arange(-0.1, 0.41, 0.1)
yticks = np.arange(-40, 41, 20)  

ax0.set_xticks(xticks)  
ax0.set_yticks(yticks)   

#plt.savefig(save_path + 'Rat_Fig3D.svg', transparent=False)
    
plt.tight_layout()
```

In [ ]:

```

```

In [ ]:

```
Hi_PE = ACC_D21[(ACC_D21.PE >0.5)]
Lo_PE = ACC_D21[(ACC_D21.PE <-0.5)]


bins = len(PE_coef)
axis1 = np.linspace(-0.1,0.4, num=bins)

fig = plt.figure(figsize=(8, 12), dpi=600)
ax0 = fig.add_subplot(311)

ax0.set_facecolor('white')

ax0.spines["top"].set_visible(False)
ax0.spines["right"].set_visible(False)

ax0.spines['left'].set_color('black')
ax0.spines['bottom'].set_color('black')
ax0.spines['left'].set_linewidth(1)
ax0.spines['bottom'].set_linewidth(1)


p1, = ax0.plot(axis1, np.mean(Hi_PE.loc[:, 'TNeg100':'T395'],axis=0), linewidth=2, color='blue', label='PE >0.5')
p2, = ax0.plot(axis1, np.mean(Lo_PE.loc[:, 'TNeg100':'T395'],axis=0), linewidth=2, color='red', label='PE <0.5')

ax0.axvline(x=0, linewidth=3, linestyle='--', color='slategray', alpha=0.5)   
ax0.axhline(y=0, linewidth=3, linestyle='--', color='slategray', alpha=0.5)   
ax0.legend(handles=[p1, p2], loc='upper right')

ax0.set_ylabel('ACC LFP activity')
ax0.set_xlabel('time surrounding tone (s)')

xticks = np.arange(-0.1, 0.41, 0.1)
yticks = np.arange(-40, 41, 20)  

ax0.set_xticks(xticks)  
ax0.set_yticks(yticks)   

#plt.savefig(save_path + 'Rat_Fig3F.svg', transparent=False)
    
plt.tight_layout()
```

In [ ]:

```

```

In [ ]:

```
def GLM_two_predictor(input_data, predictors):
        
    import statsmodels.api as sm
    import statsmodels.formula.api as smf

    activity = input_data.loc[:, 'TNeg100':'T395']

    intercept = []
    coef1 = []
    coef2 = []
    pvalues1 = []
    pvalues2 = []
    tvalues1 = []
    tvalues2 = []
    std1 = []
    std2 = []

    for g in activity:

        timebin = activity[g]
        input_data['bin'] = timebin

        md = smf.glm("bin ~ reward + Q_Chosen", input_data, family=sm.families.Gaussian())
        mdf = md.fit()

        intercept.append(mdf.params['Intercept'])
        coef1.append(mdf.params[predictors[0]])
        coef2.append(mdf.params[predictors[1]])
        pvalues1.append(mdf.pvalues[predictors[0]])
        pvalues2.append(mdf.pvalues[predictors[1]])
        tvalues1.append(mdf.tvalues[predictors[0]])
        tvalues2.append(mdf.tvalues[predictors[1]])
        std1.append(mdf.bse[predictors[0]])
        std2.append(mdf.bse[predictors[1]])
      
    return intercept, coef1, coef2, std1, std2, pvalues1, pvalues2


def plot_two_betas(betas1, betas2, pvals_corr1, pvals_corr2, plot_pvals, adjust):
    
    betas1 = np.array(betas1)
    betas2 = np.array(betas2)
    pvals_corr1 = np.array(pvals_corr1)
    pvals_corr2 = np.array(pvals_corr2)
    
    bins = len(betas1)

    axis1 = np.linspace(-0.1,0.4, num=bins)

    import matplotlib.ticker as ticker
    parameters = {'axes.labelsize': 25, 'axes.titlesize': 35, 'xtick.labelsize': 20, 'ytick.labelsize': 20}
    plt.rcParams.update(parameters)
    
    fig = plt.figure(figsize=(8, 12), dpi=600)
    ax0 = fig.add_subplot(311)

    ax0.set_facecolor('white')

    ax0.spines["top"].set_visible(False)
    ax0.spines["right"].set_visible(False)

    ax0.spines['left'].set_color('black')
    ax0.spines['bottom'].set_color('black')
    ax0.spines['left'].set_linewidth(1)
    ax0.spines['bottom'].set_linewidth(1)

    p1, = ax0.plot(axis1, betas1, linewidth=2, color='blue')
    p2, = ax0.plot(axis1, betas2, linewidth=2, color='red')

    if plot_pvals == 'yes':
        
        n_pvals = pvals_corr.shape
        max_y1 = betas.max() + adjust

        for i in pvals_corr:
            t = np.linspace(-0.1,0.4, num=bins)
            t05 = t[pvals_corr < 0.05]
            ax0.plot(t05, np.ones(t05.shape)*max_y1, '.', color='b', markersize=6)
    else:
        pass    
        
    ax0.axvline(x=0, linewidth=3, linestyle='--', color='slategray', alpha=0.5)   
    ax0.axhline(y=0, linewidth=3, linestyle='--', color='slategray', alpha=0.5)   
    
    xticks = np.arange(-0.1, 0.41, 0.1)
    yticks = np.arange(-20, 31, 10)  
    
    ax0.set_xticks(xticks)  
    ax0.set_yticks(yticks)   

    ax0.set_ylabel('regression coefficient')
    ax0.legend(handles=[p1], loc='upper right')
    ax0.set_xlabel('time surrounding tone (s)')
    
    #plt.savefig(save_path + 'Rat_SuppFig5B.svg', transparent=False)
    
    plt.tight_layout()
```

In [ ]:

```
rew_val_intercept, rew_val_coef1, rew_val_coef2, rew_val_sem1, rew_val_sem2, rew_val_pvalues1, rew_val_pvalues2 = GLM_two_predictor(ACC_D21_regression, predictors=['reward', 'Q_Chosen'])
```

In [ ]:

```
Fig3D = plot_two_betas(rew_val_coef1, rew_val_coef2,
               rew_val_pvalues1, rew_val_pvalues2,
               plot_pvals='no', 
               adjust=10)
```

In [ ]:

```

```

In [ ]:

```

```
